# Biosynthesized Silver Nanoparticles from *Eruca sativa* Miller Leaf Extract Exhibits Antibacterial, Antioxidant, Anti-Quorum-Sensing, Antibiofilm, and Anti-Metastatic Activities

**DOI:** 10.3390/antibiotics11070853

**Published:** 2022-06-25

**Authors:** Amir Mahgoub Awadelkareem, Eyad Al-Shammari, AbdElmoneim O. Elkhalifa, Mohd Adnan, Arif Jamal Siddiqui, Mitesh Patel, Mohammad Idreesh Khan, Khalid Mehmood, Fauzia Ashfaq, Riadh Badraoui, Syed Amir Ashraf

**Affiliations:** 1 Department of Clinical Nutrition, College of Applied Medical Sciences, University of Hail, Hail P.O. Box 2440, Saudi Arabia; mahgoubamir22@gmail.com (A.M.A.); eyadhealth@hotmail.com (E.A.-S.); ao.abdalla@uoh.edu.sa (A.O.E.); 2 Department of Biology, College of Science, University of Hail, Hail P.O. Box 2440, Saudi Arabia; drmohdadnan@gmail.com (M.A.); arifjamal13@gmail.com (A.J.S.); badraouir@yahoo.fr (R.B.); 3 Department of Biotechnology, Parul Institute of Applied Sciences and Centre of Research for Development, Parul University, Vadodara 391760, Gujarat, India; patelmeet15@gmail.com; 4 Department of Clinical Nutrition, College of Applied Health Sciences in Arras, Qassim University, Buraydah 58883, Saudi Arabia; moi.khan@qu.edu.sa; 5 Department of Pharmaceutics, College of Pharmacy, University of Hail, Hail P.O. Box 2440, Saudi Arabia; adckhalid@gmail.com; 6 Department of Clinical Nutrition, College of Applied Medical Sciences, Jazan University, Jazan 45142, Saudi Arabia; farooqui@jazanu.edu.sa

**Keywords:** silver nanoparticle, anticancer, antibiofilm, *Eruca sativa*, nutraceuticals, bioactive compound

## Abstract

Worldwide, the primary problem today is the proliferation of cancer and secondary bacterial infections caused by biofilms, as they are the principal causes of death due to the lack of effective drugs. A great deal of biological activities of silver nanoparticles (AgNPs) have made them a brilliant choice for the development of new drugs in recent years. The present study was conducted to evaluate the anticancer, antibacterial, anti-QS, and antibiofilm effects of AgNPs synthesized from *Eruca sativa* (*E. sativa*) leaf extract. The ultraviolet–visible (UV–Vis) spectra showed a peak of surface plasmon resonance at 424 nm λmax, which corresponded to AgNP formation. The Fourier transform infrared spectroscopy (FT-IR) confirmed that biological moieties are involved for the development of AgNPs. Moreover, transmission electron microscopy (TEM) analyses confirmed the spherical shape and uniform size (8.11 to 15 nm) of the AgNPs. In human lung cancer cells (A549), the anticancer potential of AgNPs was examined by the MTT [3-dimethylthiazol-2-yl)-2,5-diphenyltetrazolium bromide] assay, scratch assay, and invasion assay. The results indicated that AgNPs inhibit the migration of A549 cells. The synthesized AgNPs showed MIC values of 12.5 µg/mL against *Chromobacterium violaceum* (*C. violaceum*) and 25 µg/mL against *Pseudomonas aeruginosa* (*P. aeruginosa*), which demonstrated their antibacterial abilities. Biological compounds that disable the QS system are being investigated as potential strategies for preventing bacterial infections. Thus, we analyzed the potential effectiveness of synthesized AgNPs in inhibiting QS-regulated virulence factors and biofilm formation in both strains of bacteria. In *C. violaceum*, the synthesized AgNPs significantly inhibited both violacein (85.18% at 1/2 × MIC) and acyl homoserine lactone (78.76% at 1/2 × MIC). QS inhibitory activity was also demonstrated in *P. aeruginosa* at a sub-MIC concentration (1/2 × MIC) by a reduction in pyocyanin activity (68.83%), total protease (68.50%), LasA activity (63.91%), and LasB activity (56.40%). Additionally, the exopolysaccharide production was significantly reduced in both *C. violaceum* (65.79% at 1/2 × MIC) and *P. aeruginosa* (57.65% at 1/2 × MIC). The formation of biofilm was also significantly inhibited at 1/2 × MIC in *C. violaceum* (76.49%) and in *P. aeruginosa* (65.31%). Moreover, a GC–MS analysis confirmed the presence of different classes of bioactive phytochemical constituents present in the leaf extract of *E. sativa*. On the basis of our results, we conclude that biologically synthesized AgNPs showed numerous multifunctional properties and have the potential to be used against human cancer and bacterial biofilm-related infections.

## 1. Introduction

In recent years, cancer has been ranked as one of the primary causes of mortality around the world [1]. According to the reports, in the year 2020 almost 19.3 million fresh cases of cancer (19.1 million without counting nonmelanoma skin cancer) and nearly 10 million cancer deaths occurred (9.9 million without counting nonmelanoma skin cancer). Cancer deaths due to lung disease accounted for 18% of all cancer-related deaths, followed by colorectal, liver, stomach, and breast tumors [2]. Therefore, the global burden of cancer is increasing day by day and is projected to reach 28.4 million cases by the end of 2040, a 47% rise from 2020. In addition, the aggravation in cancer cases could occur due to increasing risk factors linked with urbanization and an expanding economy [3].

There are several factors that contribute to cancer’s high mortality rates, such as poor diagnosis at an early stage, and associated side effects along with the chemotherapeutic treatments [4]. As a result of chemotherapy, a patient’s immune system often becomes compromised. Patients are at risk for secondary infection from bacteria, viruses, fungi, and other pathogens. Antibiotic resistance is a concerning aspect of secondary infection in cancer patients [5]. Researchers have recently focused on developing nanoparticles (NPs) derived from metals in order to target and treat a varied range of diseases and other pathological conditions, such as cancer and secondary infections [6]. Due to favorable physico-chemical properties that can be related to biological systems, silver and gold nanoparticles are distinguished from those of noble metals [7]. Silver nanoparticles (AgNPs) are being extensively examined for their potential use as medical implants, food packaging, and environmental pollutants [8]. A number of carcinoma cell lines are reported to be cytotoxic to AgNPs, including MCF-7 (breast cancer), HeLa (cervical cancer), HT29 (colon cancer), and A549 (lung cancer) [9,10,11]. Moreover, both the scientific literature and traditional medicine have described the antimicrobial potential of AgNPs [12,13,14]. Biologically active silver ions are released during silver ionization in aqueous solution, which is responsible for AgNPs’ antimicrobial activity [12,13]. Now, scientists are focused on developing nanoparticles with certain biological activities, such as selective antibacterial, antibiofilm as well as anticancer properties. This combination of properties will be useful for treating cancer and associated infections with a single nanoparticle.

Several physico-chemical methods have been described for the synthesis of AgNPs of different types, shapes, sizes, and crystalline materials based on research and applications. Unfortunately, these physico-chemical methods are expensive, time-consuming and energy-intensive, as well as generating large quantities of toxic by-products [15,16]. The green synthesis route, on the other hand, allows AgNPs to be synthesized in a natural, economical, and environmentally friendly manner [17,18]. Plant bioactive components are a new promising source for AgNPs production rather than bacterial or chemical methods, since there is less or no risk of bacterial contamination and there is less energy consumption and higher applicability [19,20]. According to earlier reports, biologically synthesized AgNPs have been reported for their antimicrobial as well as anticancer properties [21,22].

*Eruca sativa* Miller (*E. sativa*) also known as ‘arugula, or rocket leaves’ is a nutritious salad plant of the Brassicaceae family. It is often used in food and medicine because of its aromatic qualities. The traditional use of this plant includes the stimulation of fertility and sperm production, enhancing the digestive and urinary processes, as well as fighting against eye infections [23]. The plant has recently received a lot of attention because of its high phytoactive compounds and its biological significance in various biological activities. A variety of phytochemicals are present in the plant’s various parts, including glucosinolates, tannins, phenolics, saponins, flavonoids, and essential oils [24]. The plant has been reported to possess several beneficial medicinal properties, including antimicrobial, antioxidant, anti-acne, anti-diabetic, antigenotoxic, anticancer, analgesic, anti-hyperlipidemia, anti-hyperglycemia, anti-hyperuricemia, and anti-inflammatory properties [25,26,27,28,29,30]. The leaf extract of *E. sativa* has been reported to change the silver ion oxidation state from Ag^+^ to Ag^0^ and thereby AgNPs can act as synthesizing agents without the inclusion of a reducing agent in the reaction mixture [31].

In the present study, we developed AgNPs by means of the *E. sativa* leaf extract as a bio-template to synthesize an eco-friendly and economical material under ambient conditions. To confirm the synthesis and the morphology of the generated AgNPs, their characterization was carried out via different biophysical methods. Furthermore, newly developed AgNPs were investigated for their anticancer as well as anti-metastatic potential against human lung cancer cells (A549) via the MTT (3-(4,5-dimethylthiazol2-yl)-2,5-diphenyltetrazolium bromide) assay, scratch assay, and invasion assay. The antibacterial, anti-QS and antibiofilm potential of the synthesized AgNPs was also investigated against *P. aeruginosa* and *C. violaceum*. The QS phenomenon such as violacein, AHL, pyocyanin, LasA, LasB, protease, biofilm formation and EPS productions were evaluated in the presence of *E. sativa*-capped AgNPs. Moreover, the antioxidant potential against 2,2-diphenyl-1-picryl-hydrazyl-hydrate (DPPH) and hydrogen peroxide (H_2_O_2_) radicals was evaluated, and the identification of the phytochemicals present in *E. sativa* leaf extract was also carried out via GC–MS analysis.

## 2. Results

### 2.1. Synthesis and Characterization of AgNPs

**Figure 1:**
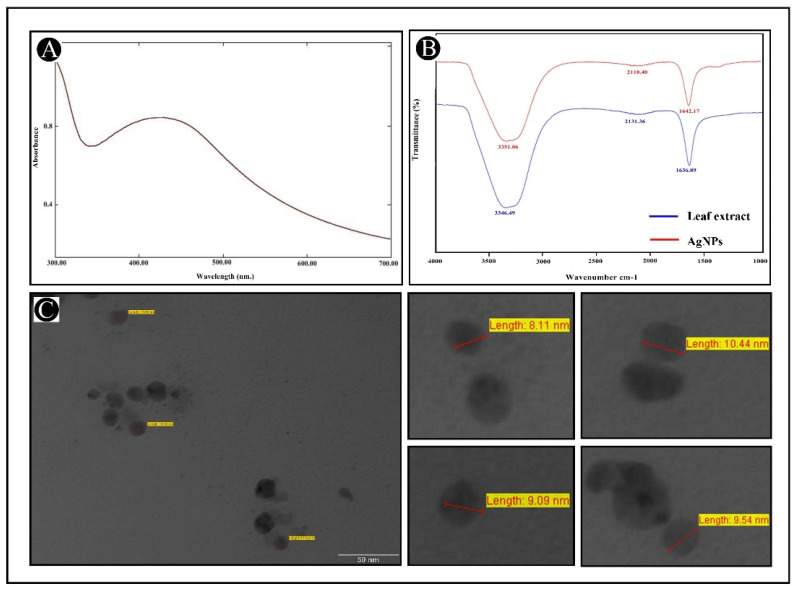
Characterization of *E. sativa*-leaf-extract-capped silver nanoparticles (AgNPs). (**A**) UV–visible absorption spectra of synthesized AgNPs. (**B**) FT-IR pattern of *E. sativa* leaf extract and synthesized AgNPs. (**C**) Morphological analysis of synthesized AgNPs with variable diameter using TEM micrograph.

The leaf extract of *E. sativa* is an inexpensive and readily available plant ingredient. We synthesized the AgNPs and then characterized them for further application in clinical, food and pharmaceutical industries, etc. UV–visible spectroscopy is a significant and extensively used system for determining the formation of AgNPs in aqueous solutions. In this study, UV–Vis analysis was used to observe the formation and stability of the AgNPs synthesized from *E. sativa* leaf extract. A spectroscopic measurement of AgNPs synthesized after 24 h revealed a clearly symmetric absorption spectrum with peak maximum at 424 nm (Figure 1A). Free electrons in AgNPs contribute to the absorption band appearance, which is caused by their mutual vibration of light wave incidences, thus causing a Surface Plasmon Resonance (SPR) absorption band. After two weeks of synthesis, spectroscopic measurements were also performed and the synthesized NPs showed no noticeable variation in the spectroscopic results, which indicates their stability. This suggests that the *E. sativa* leaf extract could be acting as a capping agent or a binding agent to provide stability to AgNPs.

FT-IR explorations were conducted to investigate the capping ability of the *E. sativa* leaf extract on AgNP surfaces. Figure 1B shows the FT-IR spectra of pure leaf extract and biosynthesized AgNPs from *E. sativa* leaf extract. The FT-IR spectrum of fresh *E. sativa* extract displayed peaks at around 3346, 2132 and 1636 cm^−1^, which correspond to the amine group’s N-H/O-H vibration stretch (around 3300 cm^−1^), C ≡ C (around 2100 cm^−1^) as well as C = C (around 1635 cm^−1^). The AgNPs synthesized using *E. sativa* extract showed peaks at around 3351, 2118 and 1642 cm^−1^, corresponding to the functional groups of amine N-H/ O-H vibration stretch (around 3370 cm^−1^), C ≡ C stretch (around 2100 cm^−1^) and amide C = O (around 1640 cm^−1^). Therefore, the FT-IR spectrum revealed that the functional groups -NH, -OH and -C = O were significant in the reduction of Ag^+^ to Ag^0^, and the widening of the peak heights in the spectrum of AgNPs authenticate that the *E. sativa* leaf extract was present on the surface of the AgNPs. It may help to stabilize the developed particles and prevent accumulation and oozing in the medium by saturating them with the extract from *E. sativa* leaves. 

Moreover, TEM images were also analyzed to investigate the detailed morphology of newly developed AgNPs. Figure 1C shows TEM images of synthesized AgNPs. The developed particles were found to be of sizes ranging from 8.11 to 15 nm. Overall, all of these results indicate that the AgNPs were prepared and stabilized successfully.

### 2.2. Anticancer Potential of Synthesized AgNPs

**Figure 2:**
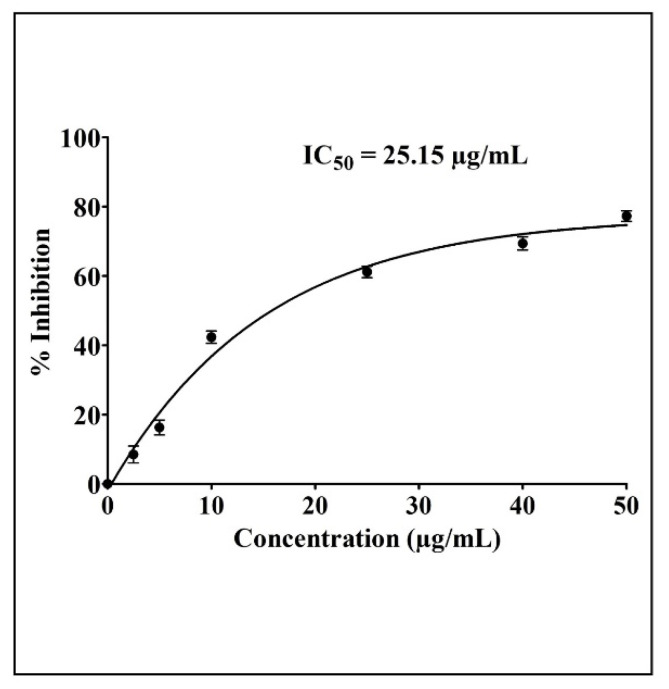
Cytotoxicity and IC_50_ (estimated value 25.15 µg/mL) of AgNPs on A549 cancer cells.

The newly developed AgNPs and their effect on the cytotoxicity of A549 cancer cells was evaluated by the MTT assay. We found that the lung cancer cell viability was considerably inhibited in a time- and concentration-dependent manner as a result of the treatment with different concentrations of AgNPs. A dose–response inhibition curve was used to determine the values of IC_50_ after 24 h and the IC_50_ value was calculated to be 25.15 µg/mL (Figure 2).

**Figure 3:**
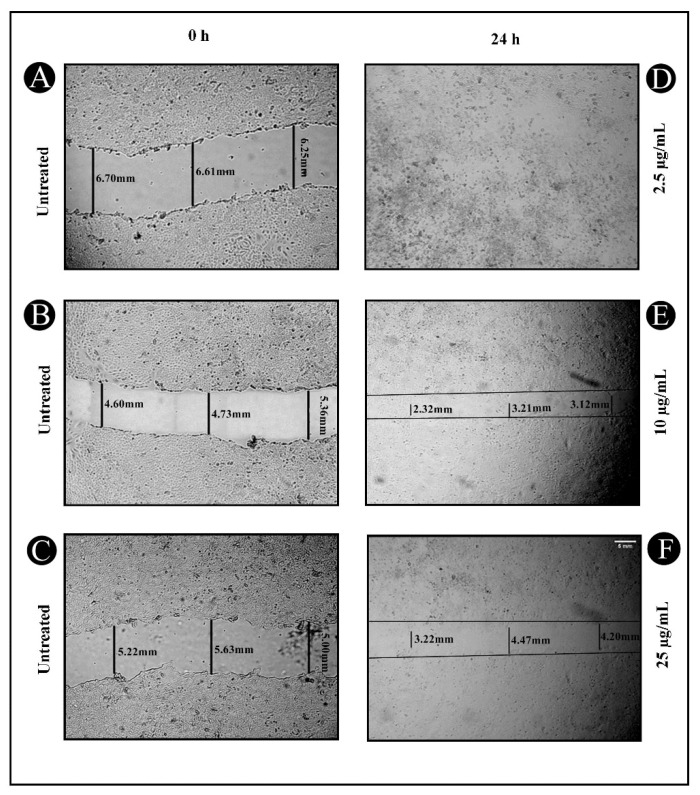
Inhibitory effect of synthesized AgNPs on A549 cell migration. (**A**–**C**) A scratch was made onto a monolayer of untreated A549 cells at 0 h. (**D**–**F**) Following treatment with different concentrations of AgNPs (2.5, 10 and 25 µg/mL) at 24 h, the migration patterns of A549 cells were recorded using inverted light microscopy.

**Figure 4:**
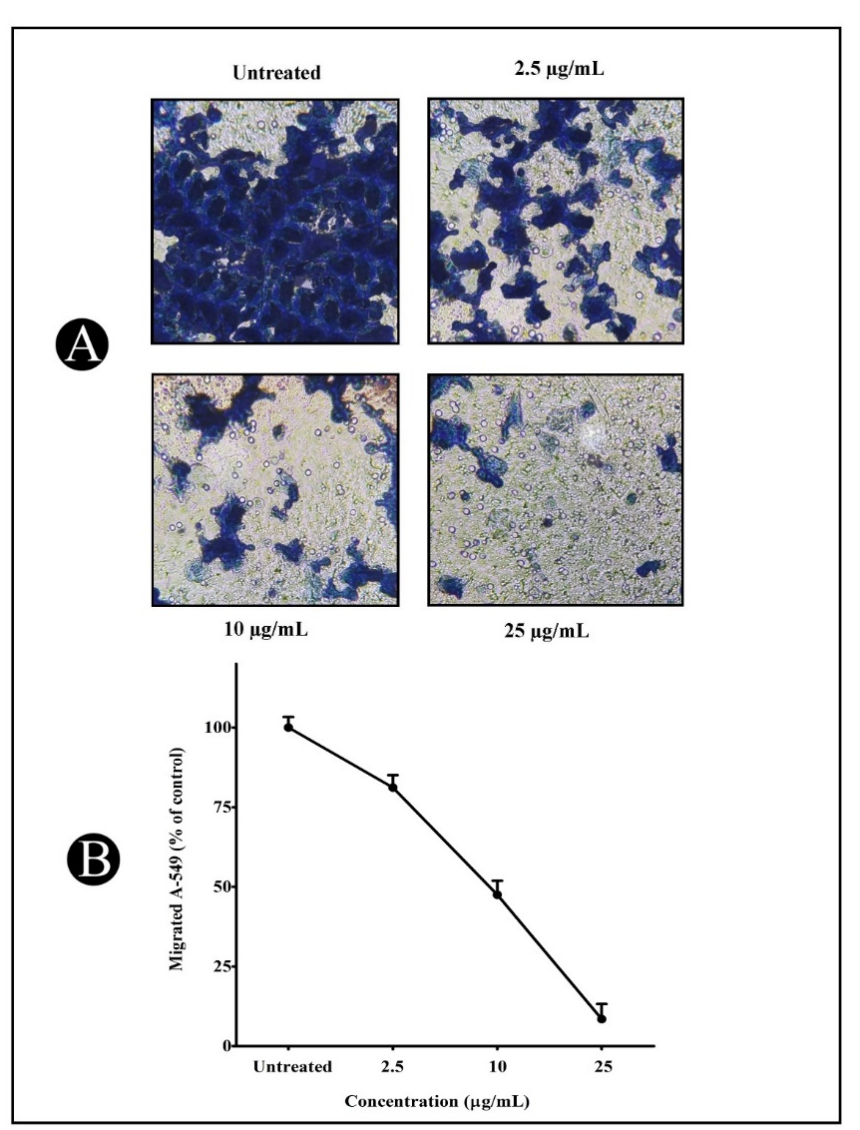
A549 cancer cell treatment with varied AgNP concentrations (2.5, 10 and 25 µg/mL) at 24 h. (**A**) Cell migration was determined using Transwell^®^ cell culture chambers (Sigma-Aldrich, Bengaluru, India), and the average number of migrated cells was determined from three randomly selected fields. (**B**) Values are calculated as a percentage of untreated controls and presented as mean ± S.D.

Cell migration is the most significant metastatic event, which occurs during the progression of cancer in various epithelial cells. Therefore, wound healing and Transwell migration tests were carried out to determine the inhibitory potential of the synthesized AgNPs on A549 cancer cell migration. As seen in Figure 3A–F, 24 h post wounding, untreated A549 cancer cells slowly migrated to the clear area, while AgNP treatment inhibited the A549 cancer cell migration. Furthermore, the outcome from the Transwell migration assay indicated that AgNP treatment might efficiently hinder the cell migration of A549 cancer cells in a concentration-dependent mode, ranging from 18.84% to 91.53%, as presented in Figure 4A,B.

### 2.3. Antibacterial Activity of Synthesized AgNPs

The investigation of the antibacterial potency of the developed AgNPs was carried out against *P. aeruginosa* and *C. violaceum*, since AgNPs are well known to have antibacterial properties against the selected pathogenic bacteria [12,13,14]. To discover the antibacterial activity of synthesized AgNPs, a broth microdilution was used to measure their MIC. The synthesized AgNPs had MIC values of 12.5 µg/mL against *C. violaceum* and 25 µg/mL against *P. aeruginosa* (Table 1).

### 2.4. Effect of Synthesized AgNPs on Quorum-Sensing (QS)-Regulated Virulence Factors

**Figure 5:**
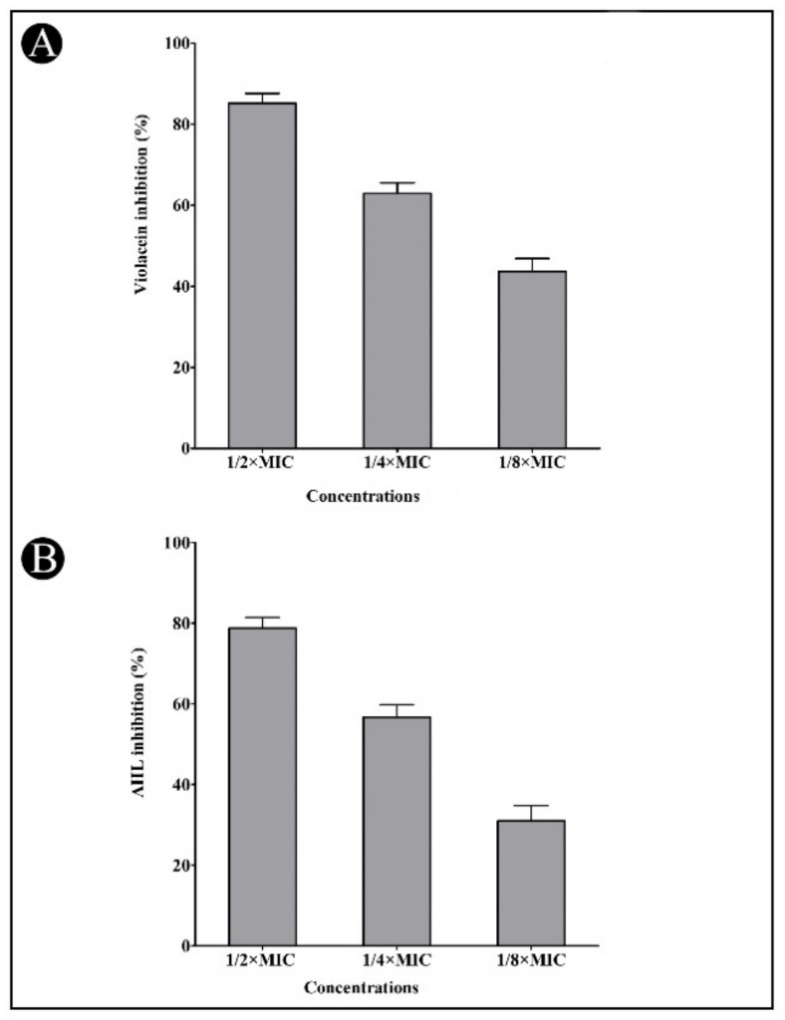
Inhibition of violacein (**A**) and AHLs (**B**) by synthesized AgNPs in *C. violaceum* at the sub-MIC concentrations (1/2, 1/4 and 1/8 MIC). Values are presented as mean ± SD of three independent tests.

The QS modulatory properties of the developed AgNPs was primarily assessed against *C. violaceum*. Upon treatment of the synthesized AgNPs, a strong inhibitory effect on pigment production was observed at a sub-MIC level, which indicates the reduction in *C. violaceum* growth, as well as anti-QS activity. The synthesized AgNPs interfered with the activity of the QS enzyme and hindered violacein pigment production in *C. violaceum*. The synthesized AgNPs reduced the violacein production by up to 85.18, 62.94 and 43.70%, respectively, at sub-MIC concentrations of 1/2 × MIC, 1/4 × MIC and 1/8 × MIC (Figure 5A). Similarly, the synthesized AgNPs inhibited AHL production in a dose-dependent manner at 78.76, 56.63, and 30.97% when compared to control at 1/2 × MIC, 1/4 × MIC and 1/8 × MIC, respectively (Figure 5B). As a result, we found that the synthesized AgNPs significantly inhibited the production of AHL when compared to the control group.

**Figure 6:**
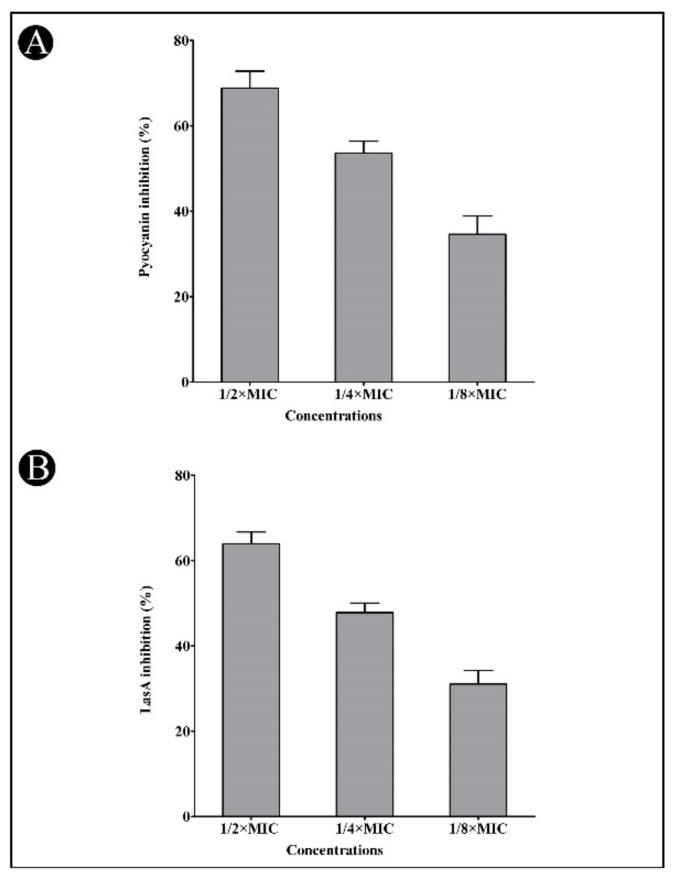
Inhibition of pyocyanin (**A**) and LasA (**B**) by synthesized AgNPs in *P. aeruginosa* at the sub-MIC concentrations (1/2, 1/4 and 1/8 MIC). Values are presented as mean ± SD of three independent tests.

A preliminary study of the synthesized AgNPs was also performed to determine their anti-QS properties in contrast to *P. aeruginosa* by scanning for virulent factors such as pyocyanin and azocasein degrading protease activity, LasA protease activity as well as LasB elastase activity. *P. aeruginosa* produces pyocyanin, which is a virulent factor that is associated with its pathogenesis and virulence. A dose-dependent reduction in pyocyanin production by the synthesized AgNPs was noted at sub-MIC concentrations (68.83, 53.60 and 34.59%, respectively) (Figure 6A). As a result of our promising pyocyanin assay results, we examined the effect of the synthesized AgNPs on *P. aeruginosa*’s LasA protease activity. Thus, we found that a reduction in LasA protease activity occurs in a concentration-dependent manner due to the occurrence of the synthesized AgNPs at sub-MIC concentrations (63.91, 47.83 and 31.08%, respectively) (Figure 6B).

**Figure 7:**
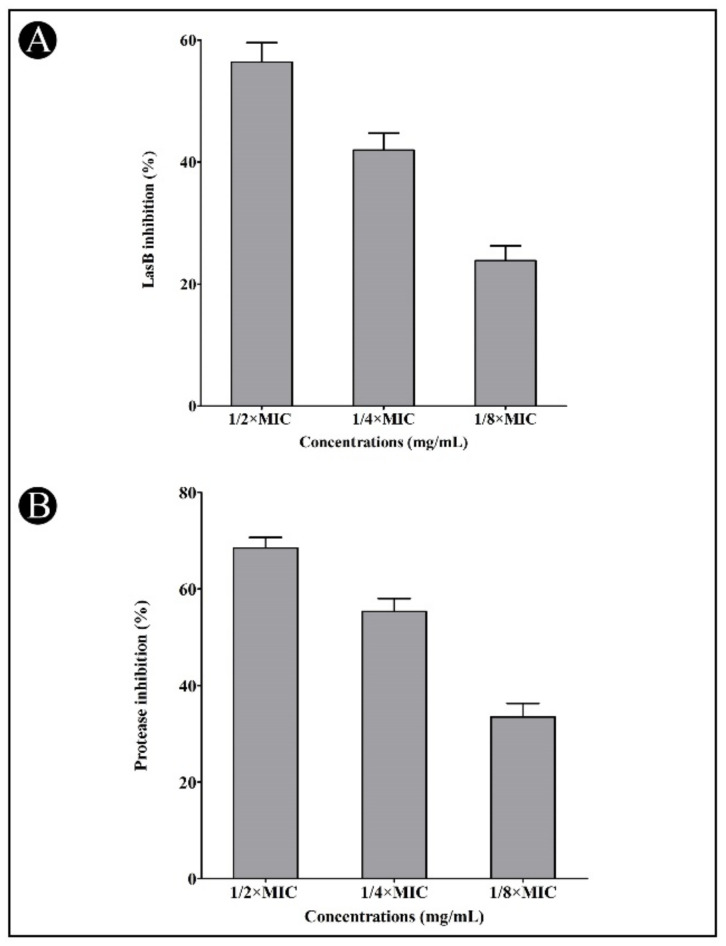
Inhibition of LasB (**A**) and protease (**B**) by synthesized AgNPs in *P. aeruginosa* at the sub-MIC concentrations (1/2, 1/4 and 1/8 MIC). Values are presented as mean ± SD of three independent tests.

**Figure 8:**
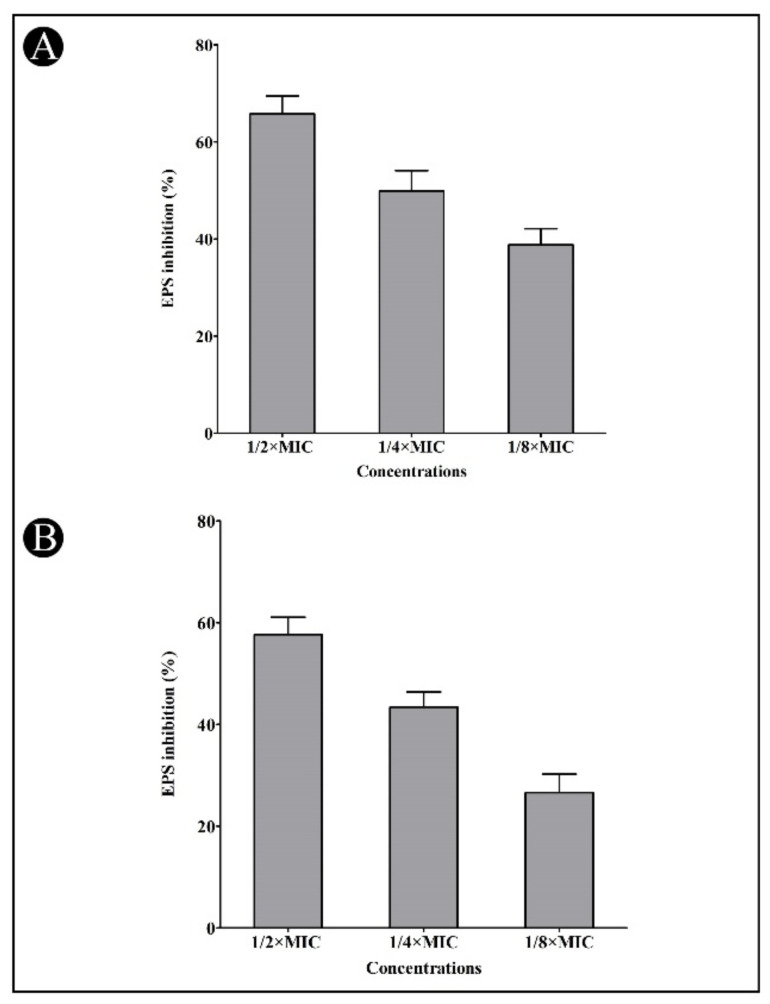
Inhibition of EPS production by synthesized AgNPs at the sub-MIC concentration. (**A**) Effect of synthesized AgNPs on EPS production in *C. violaceum* at the sub-MIC concentrations (1/2, 1/4 and 1/8 MIC). (**B**) Effect of synthesized AgNPs on EPS production in *P. aeruginosa* at the sub-MIC concentrations (1/2, 1/4 and 1/8 MIC).

In addition, LasB elastase can cause corneal and pulmonary ulcers, necrosis, and hemorrhaging, making it a particularly interesting enzyme for study. The elastase activity of LasB in *P. aeruginosa* culture supernatant was significantly reduced by the synthesized AgNPs when applied at sub-MIC concentrations (56.40, 41.95, and 23.82%, respectively) (Figure 7A). Bacteria produces proteases, which break the host cell’s proteins, allowing them to penetrate and grow. A dose-dependent suppression of bacterial proteinase production was observed using the synthesized AgNPs at sub-MIC concentrations (68.50, 55.35 and 33.47%, respectively) (Figure 7B). Biofilms comprise bacterial polymers called EPS. EPS biopolymers form a matrix in the biofilm and provide a means to retain moisture, keeping the cells together. At sub-MIC concentrations of AgNPs, the amount of EPS production was decreased in *C. violaceum* (65.79, 49.89 and 38.41%, respectively) and *P. aeruginosa* (57.65, 43.40 and 26.58%, respectively) (Figure 8A,B).

### 2.5. Antibiofilm Potential of AgNPs

**Figure 9:**
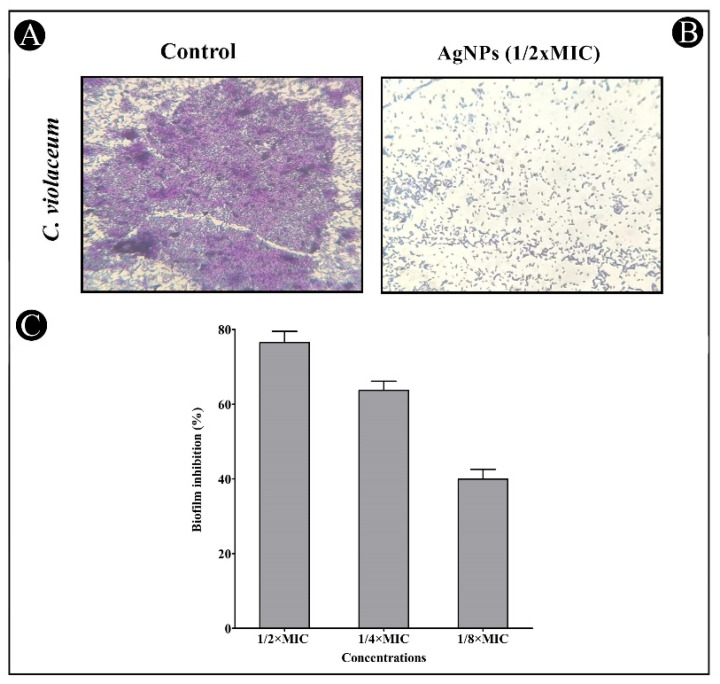
In situ light microscopy showed biofilm inhibition of synthesized AgNPs against *C. violaceum*. (**A**) Control shows biofilm formation. (**B**) The synthesized AgNPs reduced the biofilm matrix at a concentration of 1/2 MIC. (**C**) The inhibition of biofilm is presented as percentage inhibition (with respect to untreated control). Values are presented as mean ± SD of three independent tests.

**Figure 10:**
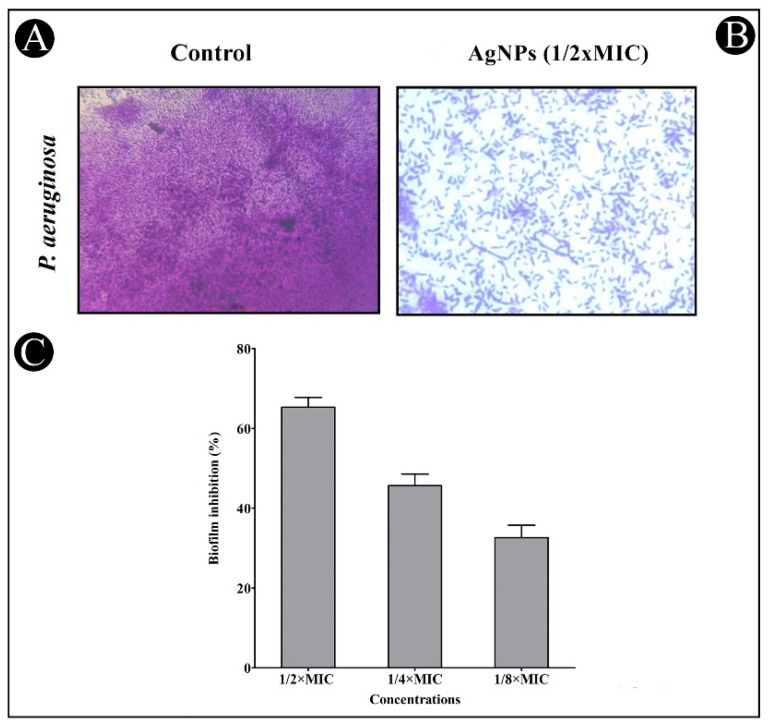
In situ light microscopy showed biofilm inhibition of the synthesized AgNPs against *P. aeruginosa*. (**A**) Control shows biofilm formation. (**B**) The synthesized AgNPs reduced the biofilm matrix at a concentration of 1/2 MIC. (**C**) The inhibition of biofilm is presented as percentage inhibition (with respect to untreated control). Values are presented as mean ± SD of three independent tests.

The crystal violet assay was used to assess AgNPs’ antibiofilm potential at sub-MIC concentrations against the tested bacterial strains. The results showed that the inhibition of both strains was concentration-dependent when using the synthesized AgNPs (Figure 9 and Figure 10). Biofilm inhibition was found to be 76.49, 63.70 and 39.94% for *C. violaceum* at sub-MIC concentrations (Figure 9C), and 65.31, 45.66 and 32.62% for *P. aeruginosa* at sub-MIC concentrations (Figure 10C). The developed AgNPs were assessed for their efficacy as an antibiofilm agent by disrupting the cell wall of bacteria using light microscopy (LM). In the presence of AgNPs, biofilms developed thinner, with fewer microcolonies, when compared with the control, which developed a dense mat of biofilms (Figure 9A,B and Figure 10A,B).

### 2.6. Antioxidant Potential of Synthesized AgNPs

**Figure 11:**
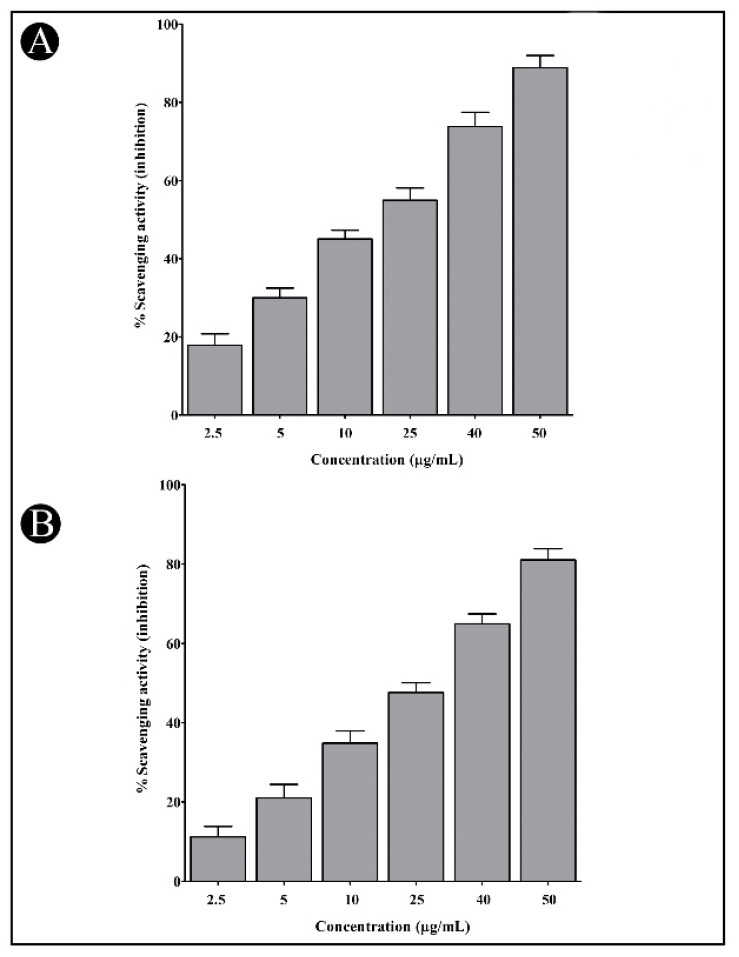
Antioxidant potential of synthesized AgNPs. (**A**) Antioxidant potential against DPPH free radical. (**B**) Antioxidant potential against H_2_O_2_ molecules. Values are presented as mean ± SD of three independent tests.

In comparison to ascorbic acid, the synthesized AgNPs were assessed for their antioxidant prospective against DPPH and H_2_O_2_ molecules. As a result, the synthesized AgNPs demonstrated good radical-scavenging capabilities against both free radicals. There was a dose dependence of the antioxidant activity of the newly developed AgNPs, indicating that with an increase in concentration (50, 40, 25, 10, 5, and 2.5 µg/mL), an increase in the antioxidant potency was also observed (Figure 11A,B).

### 2.7. Identification of Phytochemical Constituents by GC–MS

**Figure 12:**
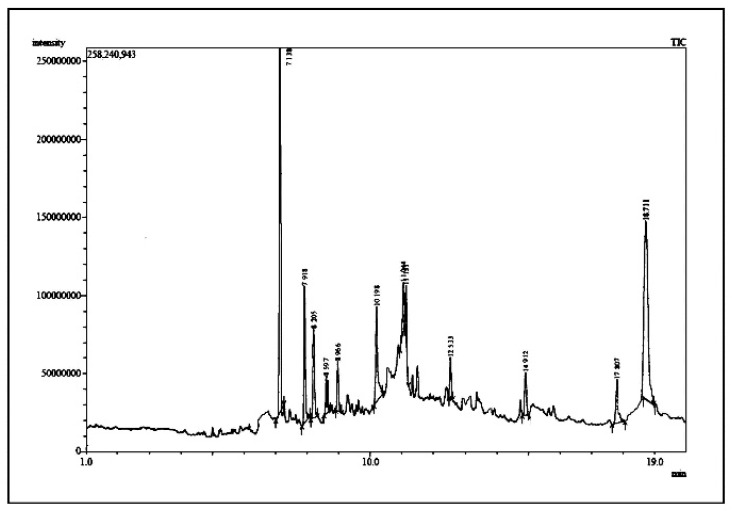
GC–MS chromatogram of *E. sativa* leaf extract.

The chemical components of the *E. sativa* leaf extract were determined by GC–MS analysis. Subsequently, all the identified chemical components are presented in Table 2. The total ion chromatogram of the leaf extract of *E. sativa* showed the occurrence of diverse biologically potent components with different retention times (Figure 12). The identified structural components were recognized by applying mass spectrometry (MS) analysis. The recognized identified chemical components were tricyclo[5.3.0.0(4,8)]decane; thiepane; 4H-Pyran-4-one,2,3-dihydro-3,5-dihydroxy-6-methyl; dodecane; D-glucitol-4-O-hexyl; tetradecane-1-iodo-; phenol,3,5-bis-1,1-dimethylethyl; sulforaphane nitrile; piperidine- 1-methanesulfonyl-4-methoxy; 9,12,15-octadecatrienoic acid; phytol and dicyclohexyl sulfide.

## 3. Discussion

NPs are smaller particles with sizes less than 100 nanometers, and since there is an increase in surface area to volume ratio of the nanoscale material/particle, nanoscale materials exhibit unique, new, and superior mechanical, as well as chemical properties compared to their bulk counterparts [32]. In general, metal NPs are studied the most since they are easier to synthesize compared to their counterparts. Furthermore, these nanoparticles have a varied range of usage in various fields, including food and pharmaceutical industries, detectors, catalysts, antibacterials, antimicrobials, and surface-coating agents. There are several metallic NPs that have been extensively studied, such as silver (Ag) [33,34] platinum (Pt) [35], gold (Au) [36], and palladium (Pd). The study of AgNPs in the areas of health and science is of particular interest. As an antibacterial, Ag is also toxic to cells. Macromolecules such as proteins and deoxyribonucleic acid (DNA) interact with Ag ions in the cells, which damage the cell wall of bacteria, inhibit bacterial cell growth, and disrupt the cell metabolism. In the interactions with the cell, the Ag ion inhibits the synthesis of proteins, further decreases the cell membrane permeability, and ultimately leads to cell death. NPs of Ag are chemically more sensitive compared to Ag in bulk. Thus, AgNPs have a greater affinity for antibacterial properties [37,38,39].

Several methods are used to synthesize AgNPs, including chemical reduction. The reduction methods are considered to be easier and more cost-effective than other methods [39]. In this method, Ag salts are reduced by reducing agents, such as sodium borohydride or sodium citrate [40]. Moreover, the application of environmentally friendly methods is highly recommended for the synthesis of AgNPs. Plants and microorganisms can be used in green synthesis techniques for the synthesis of NPs [41]. Plant secondary metabolites are utilized as reducing agents in the biosynthesis of NPs [42]. Biological agents act as stabilizers, reducers, as well as in the process of synthesizing NPs [43]. A number of plants have been used in the biosynthesis of AgNPs, and different biological activities have been evaluated.

At the present time, various types of AgNPs are developed by biological synthesis that are reported to possess various biological properties such as antimicrobial, antioxidant, antiviral and anticancer activities, etc. [44,45,46,47,48,49]. In spite of the success of many NPs synthesized using microorganisms and plants, nanoscience research is still focused on developing new NPs with specified physical, chemical, and biological properties. *E. sativa* has varieties of phytochemical constituents and is widely used in traditional medicine due to its excellent biological properties [50,51]. The present study describes the AgNP synthesis and the characterization of the *E. sativa* leaf extract that may possess biological and economic benefits. Furthermore, synthetic AgNPs have significant anticancer, anti-metastatic, antibacterial, anti-QS, antibiofilm and antioxidant activity.

The results of our study showed that AgNPs were synthesized, and when silver nitrate was exposed to the leaf extract of *E. sativa*, the alteration in color could be attributed to the formation of NPs, which were also confirmed by UV–Vis spectroscopy. As a result of its SPR properties, the biosynthesized AgNPs exhibited a strong band of absorption at 424 nm. There was a direct correlation between the amount of extract and the incubation period in this case, and it was likely due to the stimulation of longitudinal plasmon vibrations as well as the reduction of the AgNO_3_ ions [52,53]. The analysis of the FT-IR spectrum was performed to determine the key factors that contribute to the reduction of silver ions (Ag^+^) into AgNPs (Ag^0^) in the aqueous extract of *E. sativa* leaves. Comparing the absorbance peak of the AgNPs with the control leaf extract, a change in absorbance peak intensity was recorded at different points. The spectra of the leaf-extract-based AgNPs revealed a range of absorption bands between 1642 and 3351cm^−1^. Accordingly, this suggests the biomolecule’s presence and its capability of reducing and stabilizing silver ions (Ag^+^) into AgNPs (Ag^0^) present in aqueous leaf extracts. It was found that the FT-IR exploration revealed a number of absorption peaks, specifically those associated with the N-H stretching vibration, which indicates strong hydrogen bonds, and C-O extension vibrations, which are associated with ketones, carboxylic acids and aldehydes, which are related to Ag ion reduction, that resulted in the stabilization of the NPs by oxidizing the hydroxyl radical [54,55]. Based on the analysis of images obtained by TEM of the biosynthesized AgNPs, it appears that most particles were crystalline in nature and nearly spherical in shape. A few clusters of AgNPs were observed in our study, suggesting that they might be responsible for the particle size variation. Upon closer examination, it is easy to determine that the clusters are the result of individual particles clustering together. The TEM analyses of previous studies reported different sizes of spherical AgNPs containing bio-organic compounds [56,57].

Presently, cancer is still a life-threatening disease around the world despite all the progress in oncology [58]. The disease starts locally, but it could spread to other portions of the body through migration, invasion, and metastasis [59]. The process of metastasis involves a number of complex mechanisms, which include the extrication, gathering and motility of cancer cells, followed by their adhesion to endothelial cells and their growth at different sites [60]. In cancer, metastasis is the dominant cause of death because of resistance to apoptosis and cytotoxic agents. The rate of mortality and morbidity in metastatic cancer patients is high, because current chemotherapy agents do not have the ability to selectively and effectively kill the cancer cells without damaging healthy cells at the sites of metastasis [61]. Thus, around the world, metastatic disease remains a crucial clinical challenge in cancer treatment [61]. For cancer treatment, radiation therapy, chemotherapy and surgery are currently considered to be the most common methods. It is well-known that these treatments have a range of side effects on human health. As a consequence, metastasis is found to be the most challenging obstacle to successful cancer management [60].

The newly developed AgNPs were also examined for their anticancer and anti-metastatic potentials against lung cancer cells (A549) in the present study. The AgNPs showed significant cytotoxicity when tested against malignant A549 cells with an IC_50_ value of 25.15 µg/mL. Additionally, we tested the AgNPs’ effect on the migration of cells in a wound-healing assay, since migration is a key step in the development of cancer and metastasis. AgNPs inhibited the migration of cancer cells towards the wound in a significant way. The present study discovered that AgNPs could inhibit cell migration, a highly important process during the early phases of wound healing. AgNPs were also found to obstruct the invasion of malignant cancer cells. Thus, in this study, we found that AgNPs inhibited the metastatic process in A549 lung cancer cells in vitro using wound-healing and invasion assays.

We further analyzed the potency of our biosynthesized AgNPs with *E. sativa* extract targeting the biofilms and related virulence factors to confirm their activities to a broader scope. Around 65% of all bacterial infections can be attributed to biofilms [62], and as a result of the resistance of bacteria to various antibiotics, biofilm-related infections are often difficult to treat. In order to prevent the formation of biofilms, it is crucial to identify novel and effective molecules [63]. The use of antibiotics in treating biofilm-related infections is insufficient; however, understanding the mechanism of biofilm formation will facilitate the strategic control and treatment of biofilm infections [64]. The inhibition of biofilms is another way to regulate the surface-adhered bacteria’s growth and survival. Many bacterial behaviors, including virulence and biofilm formation, are influenced by a cascade of signaling reactions known as QS [65]. Bacterial pathogenicity and virulence can be reduced by obstructing the QS cascades. As a result of communicating through signal molecules, bacteria at a certain density are believed to begin expressing virulence genes, which enable the production of virulence factors. Therefore, antimicrobial therapy can now be aimed at blocking bacterial communication [66]. Antimicrobial compounds with QS inhibitory effects are new-generation antimicrobials [67]. In the fight against biofilms, AgNPs offer a new horizon due to their excellent selective and specific mechanisms of action. Considering that AgNPs synthesized from plants have a natural effect and are less toxic than anti-QS agents, they are ideal for combating pathogenic bacterial biofilms [64]. For the purpose of searching for a new method or molecule to prevent biofilm formation, we investigated AgNPs’ anti-QS and antibiofilm activity against the model pathogenic organisms *C. violaceum* and *P. aeruginosa* [57].

The purple pigment (violacein) present in *C. violaceum* makes it an ideal indicator organism for the screening of QS inhibitors. The reduction in violacein pigment production as a result of AgNPs interference with QS was evaluated in this study. Within *C. violaceum*, violacein synthesis is controlled by the CviR QS system. Consequently, any change in the amount of *C. violaceum* pigment is interpreted as a direct sign of QS hindrance. A number of natural products have also been reported to inhibit the production of violacein by *C. violaceum* [68,69]. Additionally, AgNPs inhibited AHL synthesis, indicating that AgNPs interfered with QS by inhibiting the production of AHL molecules. Based on our study of violacein and AHL inhibition, we found that AgNPs are most active. Therefore, we screened further AgNPs against *P. aeruginosa* to determine whether they have broad-spectrum QS activity, since the QS system of *P. aeruginosa* is different from *C. violaceum*.

A variety of QS-mediated phenotypes of *P. aeruginosa* were investigated using AgNPs, including azocaseinolytic activity, pyocyanin synthesis, LasA protease activity, and LasB elastase activity [70]. It has been established that virulence factors play a key role in the invasion of bacteria and their proliferation within the host population [70]. The pathogenesis of *P. aeruginosa* is caused by the secretion of proteases by this organism [70]. In the azocasein degrading assay, AgNPs were found to reduce the protease production of *P. aeruginosa* at concentrations below the MIC. Two QS systems are found in *P. aeruginosa* (LasIR and RhIIR). The first QS system (Las) occurs through the formation of diffusible signal molecules 3O-C12-HSL due to a synthase encoded by the LasI gene, which is further activated by LasR (transcriptional activator) to activate many virulence genes (LasA and LasB). Several virulent enzymes are produced by these genes, including alkaline protease, LasA protease, and LasB elastase.

*P. aeruginosa*’s second QS system (Rhl) synthesizes the diffusible signal molecules C4-HSL via RhlI, which further associates with RhlR and activates the expression system for pyocyanins. The LasIR gene encodes proteins that play an important role in the pathogenesis of *P. aeruginosa* [71]. In this study, we tested different sub-MIC concentrations of AgNPs in order to determine if they inhibited all of the QS-associated proteins and factors in a dose-dependent manner. A third and distinct virulence factor, pyocyanin, which is produced in QS, is also important and very different. Pyocyanin has been well documented as having a role in the pathogenesis of many diseases, particularly cystic fibrosis [72]. Our results are consistent with those found in the literature, where other natural products have also been reported to inhibit QS-associated proteins and factors of *P. aeruginosa* to varying degrees [73].

Synthesized AgNPs scavenge a significant amount of radicals in the antioxidant assay. A possible reason for the higher antioxidant activity of the synthesized AgNPs may lie in the presence of phytochemical components in the leaf extract and silver ions, which could result in antioxidant activity proceeding simultaneously through hydrogen atom transfer (HAT) and single electron transfer (SET) [74]. Thus, the antioxidant abilities of AgNPs recommend their use as natural antioxidants in preventing degenerative diseases caused by oxidative stress.

The medicinal value of Ag has been widely recognized since the ancient times [75]. The use of plant-based bio-constituents is currently a highly effective and eco-friendly method for manufacturing monodisperse, colloidal, homogeneous AgNP solutions. There have been several studies involving the synthesis of AgNPs from plant leaves, including *Acalypha indica* [76], *Ficus benghalensis* [76,77], and Mulberry [77,78], as well as from seed extracts of a number of plants, including *Macrotyloma uniflorum* [79], *Jatropha curcas* [80], *Illicium verum* [80,81], and *Pistacia atlantica* [75,81]. In recent years, there has been a rise in the use of nanomaterials for the advancement of diagnostic tools such as biosensing and bioimaging. The application of NPs in clinical imaging modalities could affect the biological factors in the microenvironment and be able to detect changes in the microenvironment and the smaller stage of growth of cancer or tumor cells [82]. AgNPs have also been shown to damage cells by interfering with the integrity of cell membranes, causing oxidative stress and apoptosis [83]. Therefore, in this study the antimicrobial, antibiofilm, anti-QS, antioxidant and anticancer activities of biologically synthesized AgNPs were reported. In addition, as per our literature survey, we did not find a single report of biosynthesized AgNPs that exhibited such a varied potential, which demonstrates their applicability in various biomedical applications.

## 4. Materials and Methods

### 4.1. Plant Extract Preparation and Green Synthesis of Nanoparticles

Fresh leaves of *E. sativa* were collected from the Bapalal Vaidya Botanical Research Centre, Veer Narmad South Gujarat University, Gujarat, India. Furthermore, the obtained plant material was cleaned and washed three to four times with running tap water and once with sterile distilled water (D/W). A total of 20 g of fresh leaves were finely chopped and simmered in 100 mL of distilled water for 1 h at 60 °C. Following the boiling process, the mixture was cooled and strained using Whatman filter paper number 1. After filtration, the suspension was collected and stored at 4 °C for further use as a reducer, stabilizer, and capping agent. AgNPs were prepared by mixing 5 mL of leaf extract of *E. sativa* with 45 mL of AgNO_3_ solution (0.1 M) and incubating at room temperature for a bio-reduction process with vigorous stirring. The transformation of color from transparent yellow to dark brown can be seen as a sign that AgNPs are forming.

### 4.2. Biophysical Characterization of Synthesized AgNPs

As a first step, spectrophotometric analysis was performed on the synthesized AgNPs. The reaction mixture was centrifuged at 10,000 rpm for 10 min and the obtained pellet was re-suspended in sterile D/W, which was scanned using a UV–visible spectrum with a resolution of 1 nm between 300 and 700 nm. FT-IR (Perkin-Elmer, Waltham, USA) was applied to observe the interaction between *E. sativa* leaf extract and AgNPs. FT-IR spectra were noted in the wave range of 500 to 4000 cm^−1^. TEM was also applied to quantify the size and shape of the NPs. The synthesized AgNPs (1 mL) were stained with phosphotungistic acid solution (0.5%), placed over copper grids, dried, and images were taken using TEM equipped with a CCD camera (Tecnai 20, Philips, Eindhoven, Netherlands).

### 4.3. Cytotoxic Assay (MTT Assay)

The newly developed AgNPs were tested against the human lung cancer cells (A549) for their cytotoxicity potential. In flasks (25 cm^2^), cancer cells were grown in Dulbecco’s Modified Eagle’s Medium (DMEM) (MP Biomedicals, Eschwege, Germany) with 10% Fetal Bovine Serum (FBS) and 10,000 units/mL penicillin and 5 mg/mL streptomycin antibiotic solution (Hi-Media, Bengaluru, India) at 37 °C in atmosphere humidified with 5% CO_2_. When the cells reached 80% confluence, they were seeded at a density of 10^4^ cells per well in 96-well plates and incubated in the same conditions as above. A hemocytometer was used to investigate the viability of the cells after staining with Trypan Blue (Hi-Media, Bengaluru, India) (0.4%). Afterward, cells were exposed to different concentrations of synthesized AgNPs (50 µg/mL, 40 µg/mL, 25 µg/mL, 10 µg/mL, 5 µg/mL and 2.5 µg/mL) for 24 h. The media containing AgNPs was aspirated from the plate after it was removed from the incubator. Afterwards, a medium of 200 µL containing MTT reagent (10%) (MP Biomedicals, Eschwege, Germany) was added to each well to obtain a final concentration of 0.5 mg/mL, and the plates were incubated at 37 °C for an additional 3 h under humidified atmosphere (5% CO_2_). Following this, the medium was removed and 100 µL of dimethyl sulfoxide (DMSO) (Merck, Darmstadt, Germany) was added to dissolve the formazan crystals. The absorbance of the amount of formazan crystal was measured at 570 nm and 630 nm with an ELISA reader (EL10A, Biobase, China). The percentage of growth inhibition was tabulated after deducting the background and blank, and the percent growth inhibition (IC_50_) was determined by the dose–response curve for the respective cell line. The positive control used for this assay was 5-Flourouracil (5-FU) [84].

### 4.4. Wound-Healing Assay

Using wound-healing assays, the effect of synthesized AgNPs on the cell viability of A549 cancer cells was studied. The assay was performed using monolayers of cells grown in 6-well plates. Cells were plated at a concentration of 1 × 10^6^ cells/mL for the final volume of 3 mL. In the central region of the culture, an injury line was made using a sterile 1 mL pipette tip. An inverted microscope was used to photograph three random views along each scraped line. Afterward, the cells were treated with different concentrations of AgNPs (2.5, 10 and 25 µg/mL) for another 24 h and later-on a set of images was recorded. Cell migration and wound healing are indicated by a reduction in the scraped area [85].

### 4.5. Transwell Migration Assay

A cell culture Transwell insert (8 μm pore size, 24-well format, Himedia^®^, Bengaluru, India) was used to seed 1 × 10^6^ cells in serum-free media; afterwards, cells were treated with different concentrations of AgNPs (25, 10 and 2.5µg/mL) for 24 h. The lower chamber was filled with 10% FBS. By using a cotton swab, cells that had not moved through the pores were removed after 10 h of incubation. Cells, which were pierced, were treated with methanol and stained with crystal violet (0.1%). Furthermore, the counting of cell numbers was performed in terms of mean numbers of three randomly selected fields under an inverted microscope. Afterwards, we quantified the cell numbers that had penetrated the cell membrane [86].

### 4.6. Screening of Antibacterial Activity of Synthesized AgNPs

#### 4.6.1. Bacterial Strains and Growth Conditions

Synthesized AgNPs were tested for antibacterial properties against the two bacterial strains *P. aeruginosa* (MTCC 2488) and *C. violaceum* (MTCC 2656). Both bacterial strains were grown and maintained using sterile Luria Bertani (LB) broth (HiMedia^®^, Bengaluru, India) at 30 °C in a shaking condition (120 rpm) for 24 h.

#### 4.6.2. Determination of Minimum Inhibitory Concentration (MIC)

For the determination of MIC for the newly developed AgNPs, they were tested against the selected bacterial strains, and a broth dilution method was used [87]. The inoculums were prepared from an active culture of bacteria in LB broth. Dilution of synthesized AgNPs was performed in two-fold ranging from 50 to 1.56 µg/mL and added to 96-well plates (100 µL each well). Furthermore, 20 µL of each bacterial strain culture (10^8^ CFU/mL) were incubated in their respective well for 24 h at 37 °C. Afterwards, MICs were determined as the concentration at which observable growth was inhibited. The negative control was only comprised of media, while the positive control was only comprised of bacteria inoculated without any synthesized AgNPs.

### 4.7. Quorum Sensing Inhibitory Activity in C. violaceum

#### 4.7.1. Evaluation of Anti-Quorum-Sensing (QS) Activity

For the evaluation of the anti-QS properties of the synthesized AgNPs, a well-diffusion assay was conducted using *C. violaceum*. Overnight-grown bacteria were incubated over the LB Petri plates and wells were made by using a gel puncture. In the next step, 60 µL of synthesized AgNPs (50 µg/mL) were inoculated into each punctured gel well and the plates were incubated for 24 h at 37 °C. On the next day, a zone of clearance was detected that exhibited anti-QS effects against the selected bacterial strains [88].

#### 4.7.2. Violacein Inhibition Assay

Spectrophotometric measurements of *C. violaceum* produced violacein pigment were performed in the absence, as well as in the presence of synthesized AgNPs [89]. Briefly, 16–18 h (OD600 nm = 0.1) grown cultures were placed into Erlenmeyer flasks filled with LB broth along with the presence or absence of synthesized AgNPs (sub-MIC—1/2, 1/4 and 1/8 MIC) and incubated for 24 h at 28 °C. Once incubation was completed, colonies of bacterial cells were collected via centrifugation (10,000 rpm for 10 min) and dissolved in 1 mL of DMSO. Afterwards, centrifugation was again carried out under the same conditions that were used in the previous step to remove dead cells, and the absorbance of soluble violacein was observed at 585 nm. Analyzing the OD at 585 nm, a comparison between the amount of treated *C. violaceum* and the untreated control was carried out. The magnitude of the decline in violacein production in presence of synthesized AgNPs was determined by using the formula presented below

Violacein inhibition (%) = [Control (OD) − Treated (OD)/Control (OD)] × 100

#### 4.7.3. Quantification of Acyl Homoserine Lactones (AHLs)

The AHLs of *C. violaceum* treated with or without synthesized AgNPs were evaluated using the Taghadosi et al. (2015) method with slight modifications [90]. First, 20 µL of *C. violaceum* culture were mixed with 180 µL of LB medium either with (sub-MIC—1/2, 1/4 and 1/8 MIC) or without synthesized AgNPs, and were incubated at 30 °C for 24 h under shaking conditions. After incubation, the cell supernatants were transferred to a new tube and equal volumes of acidified ethyl acetate (0.5% glacial acetic acid) were added and mixed. A rotary evaporator at 45 °C was then used to dry the organic layer collected from control and treatment tubes. In order to re-use the residues, the residues were suspended in 20% acidified ethyl acetate and kept under refrigeration at −20 °C. Residues from the treated and control experiments were placed in the wells and mixed with equal volumes of (2 M) hydroxylamine/NaOH (3.5 M). Following this, equal volumes of ferric chloride (4 M HCl (10%)) and 95% ethanol were also added, and then the absorbance of the samples was recorded at 520 nm.

### 4.8. Quorum-Sensing Inhibitory Activity in P. aeruginosa

#### 4.8.1. Quantitative Analysis of Pyocyanin Production in *P. aeruginosa*

Extracted pyocyanins from the culture supernatants of *P. aeruginosa* were obtained according to the method referred by Essar et al. (1990) [91], and were treated with or without synthesized AgNPs. Firstly, the 5 mL supernatant of *P. aeruginosa* treated with (sub-MIC—1/2, 1/4 and 1/8 MIC) or without synthesized AgNPs was extracted with 3 mL chloroform and then re-extraction was performed using HCl of 0.2 M (1 mL). Afterwards, the obtained solution was poured into a glass cuvette for measuring the absorbance at 520 nm.

#### 4.8.2. LasA Staphylolytic Assay

A boiled *S. aureus* cell suspension was lysed with *P. aeruginosa* culture supernatant to evaluate the LasA protease activity [71]. A culture of *S. aureus* cells (10^6^ CFU/mL) that was grown overnight was centrifuged at 8000 rpm for 5 min to collect the cell pellet. Collected cell pellets were suspended in 0.02 M Tris–HCl (pH 8.5) and boiled for 10 min. After boiling, the cell suspension was diluted with the same buffer to make an optical density of 0.8 at 595 nm. Then, the cell-free culture supernatants of *P. aeruginosa*, cultivated with sub-MIC concentrations of (1/2, 1/4 and 1/8 MIC) or without synthesized AgNPs, were added to diluted *S. aureus* suspensions (9:1). In the subsequent step, the solution was poured into a cuvette for measuring the absorbance at 595 nm.

#### 4.8.3. LasB Elastase Assay

LasB elastase activity was measured as per the method described by Adonizio et al. (2008) [70] to determine the elastolytic activity. A culture of *P. aeruginosa* was incubated with synthesized AgNPs (sub-MIC—1/2, 1/4 and 1/8 MIC). The treated and non-treated cultures were incubated in 900 µL elastin Congo Red buffer (100 mM Tris, 1mM CaCl_2_, pH 7.5) holding 20 mg of elastin Congo Red (Sigma, Bengaluru, India). A centrifuge was used to remove the insoluble Congo Red elastin after 3 h at 37 °C in a shaker incubator. Afterwards, the collected supernatant absorbance was recorded at 495 nm. The LB medium without synthesized AgNPs was used as a negative control.

#### 4.8.4. Azocasein Assay for Proteolytic Activity

The proteolytic activity of *P. aeruginosa* supernatants cultured with (sub-MIC—1/2, 1/4 and 1/8 MIC) or without synthesized AgNPs was determined by applying the method referred by Kessler et al. (1993) [71]. Afterwards, 150 µL of culture supernatants were added to 1 mL (0.3%) of azocasein (Sigma, St. Louis, USA) in 0.05 M Tris–HCl and 0.5 mM CaCl_2_ (pH 7.5), and the incubation of supernatants was performed at 37 °C for 15 min. To stop the reaction, 0.5 mL of 10% trichloroacetic acid was added. After centrifugation, the supernatant was collected and absorbance was measured at 400 nm.

#### 4.8.5. Extraction and Estimation of Exopolysaccharides (EPS)

Both *C. violaceum* and *P. aeruginosa* bacterial strains were grown in the presence of synthesized AgNPs (sub-MIC—1/2, 1/4, and 1/8 MIC) and then centrifuged and filter-sterilized. EPS was precipitated overnight at 4 °C via the addition of chilled absolute ethanol to the supernatant [92].

### 4.9. Antibiofilm Assay

The antibiotic potential of synthetic AgNPs at sub-MIC were evaluated using the crystal violet staining method [93]. Bacterial cell cultures incubated in 96-well microtiter plates using glucose (0.2%)-supplemented LB (100 µL) of each test strain (10^8^ CFU/mL) and the synthesized AgNPs were incubated at 37 °C for 24 h. Afterwards, planktonic cells were carefully removed from the wells immediately after incubation and washed with PBS (200 µL). In order to visualize biofilms, attached cells were stained for 30 min with crystal violet (0.1%) and incubated at 37 °C. Then, PBS was used to wash off excess dye, and 200 µL of 95% ethanol was used to fix the plates and the absorbance of samples were measured using a spectrophotometer at 470 nm.

#### In Situ Visualization of Biofilms

To assess the biofilms formed on glass cover slips by tested strains, we referred to a method designated by Musthafa et al. (2010) [94]. First, 24-well microtiter plates consisting of 1 × 1 cm cover slips were inoculated with 500 µL of the selected cultures in glucose (0.2%)-supplemented LB with the concentrations of 10^8^ CFU/mL. The same well was treated with 500 µL of synthesized AgNPs (sub-MIC). After 24 h of incubation at 37 °C, glass cover slips containing biofilms were gently removed and washed with PBS. Biofilms were stained with 0.1% crystal violet and observed under LM with a magnification of 40× (Axioscope A1, ZEISS, Jena, Germany).

### 4.10. Determination of DPPH Free-Radical-Scavenging Activity

The ability of synthesized AgNPs to scavenge radicals was measured in terms of their antioxidant activity against DPPH. Different concentrations of synthesized AgNPs, i.e., 50, 40, 25, 10, 5, 2.5 µg/mL were mixed with 2 mL of 6 × 10^−5^ M of DPPH solution in DMSO. Tubes were incubated in the dark for up to an hour. Afterwards, sample absorbance was measured at 517 nm. DPPH solution without AgNPs was used as a control. DMSO aqueous solutions and ascorbic acid were used as a blank and standard, respectively [50]. The DPPH radical-scavenging ability was determined using the equation below:

DPPH scavenging activity (%) = (A0 − A1)/A0 × 100

where, A0 = absorbance of the control; A1 = absorbance of the sample.

### 4.11. Determination of Hydrogen Peroxide Scavenging Activity

The hydrogen peroxide (H_2_O_2_) scavenging activity of synthesized AgNPs was measured using the method presented by Ruch et al. [95]. Each of the different concentrations of AgNPs (50, 40, 25, 10, 5 and 2.5 µg/mL) was mixed with an H_2_O_2_ solution (2 mM, 1 mL) prepared in phosphate buffer (0.1 M, pH 7.4) and incubated for 10 min at room temperature. In order to determine the absorbance at 230 nm, phosphate buffer without H_2_O_2_ was used as a blank and ascorbic acid solution was used as a positive control. The following formula was used to calculate how much H_2_O_2_ was scavenged:

% inhibition = (A0 − A1)/A0) × 100

where A0 is the absorbance of the control; A1 is the absorbance of the extract/standard.

### 4.12. Gas Chromatography-Mass Spectrophotometry (GC–MS) Analysis

In order to analyze the *E. sativa* leaf extract by GC–MS, we used a Shimadzu Nexis GC-2030 Gas Chromatograph (GC) coupled with a QP2020 NX Mass Spectrometer. For the separation of the analytes, the column temperature was adjusted to 50 °C for 3 min, raised at a frequency of 10 °C per minute up to 270 °C, and finally raised to 300 °C for 10 min. The system was filled with 10 µL of sample and the carrier gas that was used was helium. To determine the probable composition of the leaf extract, the peaks obtained from the GC–MS separation were compared with the NIST database [96].

## 5. Conclusions

The development of a reliable and eco-friendly method for producing metallic nanoparticles is a crucial aspect of nanotechnology. Currently, nanoparticles are regarded as fundamental components of nanotechnology. The physiochemical properties of silver nanoparticles make them a very attractive substance to be used in the fields of biology and medicine. Using a natural and low-cost biological reduction agent in conjunction with leaf extracts of *E. sativa*, we were able to demonstrate in the present study that a green nano-chemistry methodology can be effectively and efficiently utilized to produce metal nanostructures, thus eluding the usage of toxic solvents and the creation of waste. As tested by MTT, the synthesized AgNPs displayed significant anticancer activity against A549 cancer cells. The scratch assay and invasion assay also demonstrated the inhibitory effect on cancer cell migration. In addition, these AgNPs exhibited excellent antibacterial, anti-QS, and antibiofilm properties against *C. violaceum* and *P. aeruginosa*. The prepared AgNPs showed anti-QS activity against *C. violaceum* by inhibiting violacein and acyl homoserine lactone and against *P. aeruginosa* via a reduction in pyocyanin activity, total protease, LasA activity, and LasB activity. Furthermore, there was a significant decline in EPS production in the presence of AgNPs. The initial attachment and maturation of biofilms are based on these factors. Therefore, it is possible to further evaluate the AgNPs synthesized in this study for their anticancer and antibiofilm activities and optimize them for industrial scale production. It would be worthwhile to carry out more research on the efficacy and dose response of *E. sativa* AgNPs for use in clinical applications. Further, these AgNPs can be used in developing nutraceuticals, drugs and fabricating medical devices such as catheters, probes, etc. that resist colonization by pathogenic bacteria resistant to antibiotics.

## Figures and Tables

**Table 1:** AgNPs minimum inhibitory concentrations against pathogenic bacteria.

Bacterial Strains	MIC of AgNPs (µg/mL)	Sub MICs of AgNPs Selected for Assays (µg/mL)		
		½ MIC	¼ MIC	1/8 MIC
*C. violaceum*	12.5	6.25	3.12	1.56
*P. aeruginosa*	25	12.5	6.25	3.12

**Table 2:** GC–MS analysis of *E. sativa* leaf extract.

Compounds	RT (min.)	Molecular Formula	Structure	Fragmentation
Tricyclo[5.3.0.0(4,8)]decane	7.138	C_10_H_16_	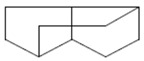	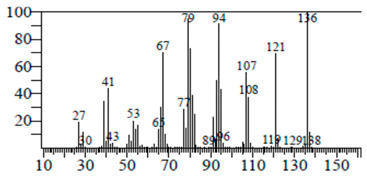
Thiepane	7.918	C_4_H_4_S	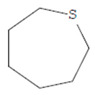	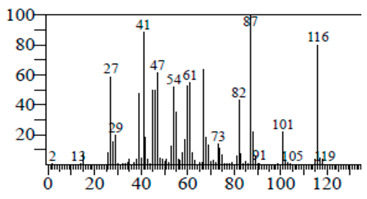
4H-Pyran-4-one, 2,3-dihydro-3,5	8.205	C_6_H_8_O_4_	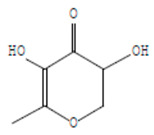	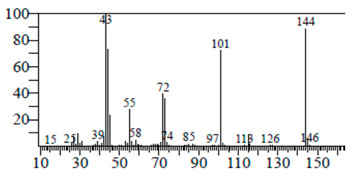
Dodecane	8.597	C_12_H_26_	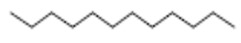	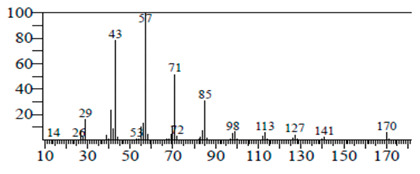
D-Glucitol, 4-O-hexyl-	8.966	C_18_H_26_O_12_	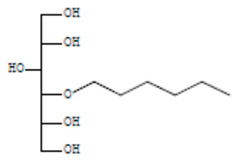	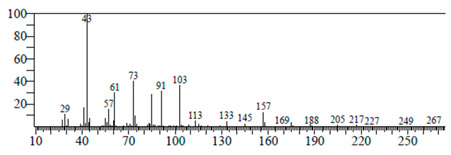
Tetradecane, 1-iodo-	10.198	C_14_H_29_I	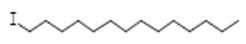	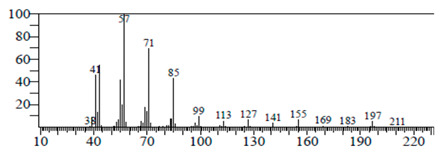
Phenol, 3,5-bis(1,1-dimethylethyl	11.044	C_27_H_50_OP_2_	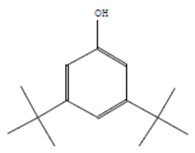	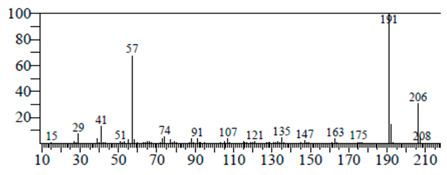
Sulforaphane nitrile	11.131	C_6_H_11_NOS_2_	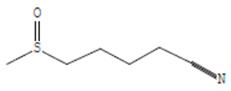	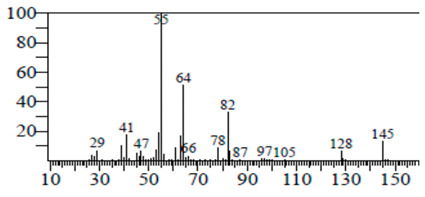
Piperidine, 1-methanesulfonyl-4-methoxy	12.533	C_13_H_19_NO_3_S	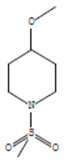	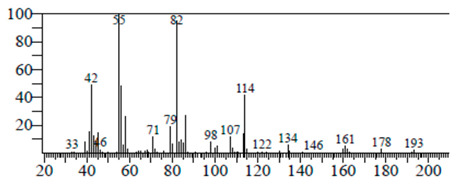
9,12,15-Octadecatrienoic acid	14.912	C_18_H_30_O_2_	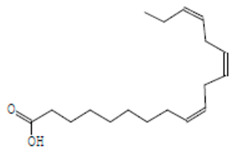	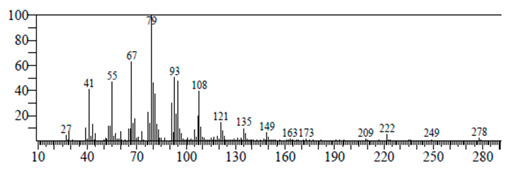
Phytol	17.807	C_20_H_40_O	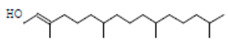	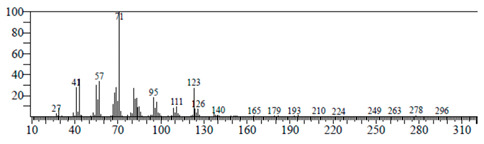
Dicyclohexyl sulfide	18.711	C_12_H_22_S_2_	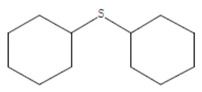	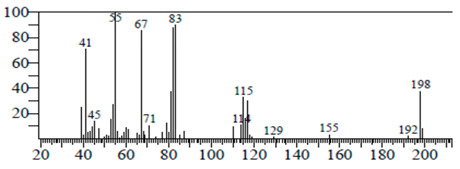

## Data Availability

All data generated or analyzed during this study are included in this article.

## References

[1] Bray F., Laversanne M., Weiderpass E. (2021). The ever-increasing importance of cancer as a leading cause of premature death worldwide. Cancer.

[2] World Health Organization (2020). Global Health Estimates 2020: Deaths by Cause, Age, Sex, by Country andby Region, 2000–2019.

[3] Sung H., Ferlay J., Siegel R.L. (2021). Global Cancer Statistics 2020: GLOBOCAN Estimates of Incidence and Mortality Worldwide for 36 Cancers in 185 Countries. CA Cancer J. Clin..

[4] Siegel R.L., Miller K.D., Jemal A. (2017). Cancer Statistics, 2017. CA Cancer J. Clin..

[5] Muteeb G., Rehman M.T., Ali S.Z., Al-Shahrani A.M., Kamal M.A., Ashraf G.M. (2017). Phage Display Technique: A Novel Medicinal Approach to Overcome an tibiotic Resistance by Using Peptide-Based Inhibitors Against β-Lactamases. Curr. Drug Metab..

[6] Oves M., Aslam M., Rauf M.A., Qayyum S., Qari H.A., Khan M.S., Alam M.Z., Tabrez S., Pugazhendhi A., Ismail I.M.I. (2018). Antimicrobial and anticancer activities of silver nanoparticles synthesized from the root hair extract of *Phoenix dactylifera*. Mater. Sci. Eng. C Mater. Biol. Appl..

[7] Mody V.V., Siwale R., Singh A., Mody H.R. (2010). Introduction to metallic nanoparticles. J. Pharm. Bioallied Sci..

[8] Calderón-Jiménez B., Johnson M.E., Montoro Bustos A.R., Murphy K.E., Winchester M.R., Vega Baudrit J.R. (2017). Silver Nanoparticles: Technological Advances, Societal Impacts, and Metrological Challenges. Front. Chem..

[9] Gengan R.M., Anand K., Phulukdaree A., Chuturgoon A. (2013). A549 lung cell line activity of biosynthesized silver nanoparticles using *Albizia adianthifolia* leaf. Colloids Surfaces. B Biointerfaces.

[10] Jeyaraj M., Rajesh M., Arun R., MubarakAli D., Sathishkumar G., Sivanandhan G., Dev G.K., Manickavasagam M., Premkumar K., Thajuddin N. (2013). An investigation on the cytotoxicity and caspase-mediated apoptotic effect of biologically synthesized silver nanoparticles using *Podophyllum hexandrum* on human cervical carcinoma cells. Colloids Surfaces. B Biointerfaces.

[11] Sanpui P., Chattopadhyay A., Ghosh S.S. (2011). Induction of apoptosis in cancer cells at low silver nanoparticle concentrations using chitosan nanocarrier. ACS Appl. Mater. Interfaces.

[12] Choi O., Deng K.K., Kim N.J., Ross L., Surampalli R.Y., Hu Z. (2008). The inhibitory effects of silver nanoparticles, silver ions, and silver chloride colloids on microbial growth. Water Res..

[13] Rai M., Yadav A., Gade A. (2009). Silver nanoparticles as a new generation of antimicrobials. Biotechnol. Adv..

[14] Zhang Y., Yang D., Kong Y., Wang X., Ginoble Pandoli O., Gao G. (2010). Synergetic Antibacterial Effects of Silver Nanoparticles@Aloe Vera Prepared via a Green Method. Nano Biomed. Eng..

[15] Mishra S., Singh B.R., Naqvi A.H., Singh H.B. (2017). Potential of biosynthesized silver nanoparticles using *Stenotrophomonas* sp. BHU-S7 (MTCC 5978) for management of soil-borne and foliar phytopathogens. Sci. Rep..

[16] Bagheri H., Banihashemi S. (2015). Sol-gel-based silver nanoparticles-doped silica—Polydiphenylamine nanocomposite for micro-solid-phase extraction. Anal. Chim. Acta.

[17] Ueno S., Nakashima K., Sakamoto Y., Wada S. (2015). Synthesis of Silver-Strontium Titanate Hybrid Nanoparticles by Sol-Gel-Hydrothermal Method. Nanomaterials.

[18] Ali K., Ahmed B., Dwivedi S., Saquib Q., Al-Khedhairy A.A., Musarrat J. (2015). Microwave Accelerated Green Synthesis of Stable Silver Nanoparticles with Eucalyptus globulus Leaf Extract and Their Antibacterial and Antibiofilm Activity on Clinical Isolates. PLoS ONE.

[19] Maddinedi S.B., Mandal B.K., Maddili S.K. (2017). Biofabrication of size controllable silver nanoparticles—A green approach. J. Photochem. Photobiol. B Biol..

[20] Azmath P., Baker S., Rakshith D., Satish S. (2017). Corrigendum to “Mycosynthesis of silver nanoparticles bearing antibacterial activity”. Saudi Pharm. J..

[21] Venkatesan J., Kim S.K., Shim M.S. (2016). Antimicrobial, Antioxidant, and Anticancer Activities of Biosynthesized Silver Nanoparticles Using Marine Algae *Ecklonia cava*. Nanomaterials.

[22] Kanjikar A.P., Hugar A.L., Londonkar R.L. (2018). Characterization of phyto-nanoparticles from *Ficus krishnae* for their antibacterial and anticancer activities. Drug Dev. Ind. Pharm..

[23] Gulfraz M., Sadiq A., Tariq H., Imran M., Qureshi R. (2011). Phytochemical analysis and antibacterial activity of *Eruca sativa* seed. Pak. J. Bot..

[24] Di Gioia F., Avato P., Serio F., Argentieri M.P. (2018). Glucosinolate profile of *Eruca sativa*, *Diplotaxis tenuifolia* and *Diplotaxis erucoides* grown in soil and soilless systems. J. Food Compos. Anal..

[25] Alqasoumi S., Al-Sohaibani M., Al-Howiriny T., Al-Yahya M., Rafatullah S. (2009). Rocket “Eruca sativa”: A salad herb with potential gastric anti-ulcer activity. World J. Gastroenterol..

[26] Zuhair T. (2016). Phytochemicals Screening by GC/MS and Determination of Some Flavonol in Cultivated Iraqi *Eruca sativa* Dried Leaves Extract and its Biological Activity as Antioxidant. Int. J. Pharmacogn. Phytochem. Res..

[27] Nurzyńska-Wierdak R. (2015). Nutritional and energetic value of *Eruca sativa* Mill. leaves. Acta Sci. Pol. Hortorum Cultus.

[28] Jirovetz L., Smith D., Buchbauer G. (2002). Aroma compound analysis of *Eruca sativa* (Brassicaceae) SPME headspace leaf samples using GC, GC-MS, and olfactometry. J. Agric. Food Chem..

[29] Nazif N.M., Habib A.A.E., Tawfik W.A.M., Hassan R.A. (2010). Chemical composition and cytotoxic activity of *Eruca sativa* L. Seeds cultivated in Egypt. Asian J. Chem..

[30] Hussein S.A. (2014). Phytochemical Study of Vsome Medicinal Compounds Present in *Hedera helix* L. Plant Cultivated in Iraq. Master’s Thesis.

[31] Alaraidh I.A., Ibrahim M.M., El-Gaaly G.A. (2014). Evaluation of Green Synthesis of Ag Nanoparticles Using *Eruca sativa* and *Spinacia oleracea* Leaf Extracts and Their Antimicrobial Activity. Iran. J. Biotechnol..

[32] Cushing B.L., Kolesnichenko V.L., O’Connor C.J. (2004). Recent Advances in the Liquid-Phase Syntheses of Inorganic Nanoparticles. Chem. Rev..

[33] Ajitha B., Ashok Kumar Reddy Y., Sreedhara Reddy P. (2015). Green synthesis and characterization of silver nanoparticles using *Lantana camara* leaf extract. Mater. Sci. Eng. C Mater. Biol. Appl..

[34] Feng A., Wu S., Senyuan C., Zhang H., Shao W., Xiao Z. (2013). Synthesis of Silver Nanoparticles with Tunable Morphologies via a Reverse Nano-Emulsion Route. Mater. Trans..

[35] Aritonang H.F., Onggo D., Ciptati C., Radiman C.L. (2014). Synthesis of Platinum Nanoparticles from K_2_PtCl_4_ Solution Using Bacterial Cellulose Matrix. J. Nanoparticles.

[36] Dos Santos M.M., Queiroz M.J., Baptista P.V. (2012). Enhancement of antibiotic effect via gold:silver-alloy nanoparticles. J. Nanoparticle Res..

[37] Vazquez-Muñoz R., Borrego B., Juárez-Moreno K., García-García M., Mota Morales J.D., Bogdanchikova N., Huerta-Saquero A. (2017). Toxicity of silver nanoparticles in biological systems: Does the complexity of biological systems matter?. Toxicol. Lett..

[38] Kim J., Kwon S., Ostler E. (2009). Antimicrobial effect of silver-impregnated cellulose: Potential for antimicrobial therapy. J. Biol. Eng..

[39] Zielińska A., Skwarek E., Zaleska A., Gazda M., Hupka J. (2009). Preparation of silver nanoparticles with controlled particle size. Procedia Chem..

[40] Sondi I., Salopek-Sondi B. (2004). Silver nanoparticles as antimicrobial agent: A case study on *E. coli* as a model for Gram-negative bacteria. J. Colloid Interface Sci..

[41] Mulfinger L., Solomon S., Bahadory M., Jeyarajasingam A., Rutkowsky S., Boritz C. (2007). Synthesis and Study of Silver Nanoparticles. J. Chem. Educ..

[42] Jirovetz L., Buchbauer G., Shafi M.P., Leela N.K. (2003). Analysis of the essential oils of the leaves, stems, rhizomes and roots of the medicinal plant *Alpinia galanga* from southern India. Acta Pharm..

[43] Chandran S.P., Chaudhary M., Pasricha R., Ahmad A., Sastry M. (2006). Synthesis of gold nanotriangles and silver nanoparticles using *Aloe vera* plant extract. Biotechnol. Prog..

[44] Kim J.S., Kuk E., Yu K.N., Kim J.-H., Park S.J., Lee H.J., Kim S.H., Park Y.K., Park Y.H., Hwang C.-Y. (2007). Antimicrobial effects of silver nanoparticles. Nanomed. Nanotechnol. Biol. Med..

[45] Rogers J.V., Parkinson C.V., Choi Y.W., Speshock J.L., Hussain S.M. (2008). A Preliminary Assessment of Silver Nanoparticle Inhibition of Monkeypox Virus Plaque Formation. Nanoscale Res. Lett..

[46] Galdiero S., Falanga A., Vitiello M., Cantisani M., Marra V., Galdiero M. (2011). Silver nanoparticles as potential antiviral agents. Molecules.

[47] Oves M., Khan M.S., Zaidi A., Ahmed A.S., Ahmed F., Ahmad E., Sherwani A., Owais M., Azam A. (2013). Antibacterial and cytotoxic efficacy of extracellular silver nanoparticles biofabricated from chromium reducing novel OS4 strain of *Stenotrophomonas maltophilia*. PLoS ONE.

[48] Bhuyan T., Mishra K., Khanuja M., Prasad R., Varma A. (2015). Biosynthesis of zinc oxide nanoparticles from *Azadirachta indica* for antibacterial and photocatalytic applications. Mater. Sci. Semicond. Process..

[49] Aziz N., Pandey R., Barman I., Prasad R. (2016). Leveraging the Attributes of Mucor hiemalis-Derived Silver Nanoparticles for a Synergistic Broad-Spectrum Antimicrobial Platform. Front. Microbiol..

[50] Awadelkareem A.M., Al-Shammari E., Elkhalifa A.E.O. (2022). Phytochemical and In Silico ADME/Tox Analysis of *Eruca sativa* Extract with Antioxidant, Antibacterial and Anticancer Potential against Caco-2 and HCT-116 Colorectal Carcinoma Cell Lines. Molecules.

[51] Awadelkareem A.M., Al-Shammari E., Elkhalifa A.O. (2022). Anti-Adhesion and Antibiofilm Activity of *Eruca sativa* Miller Extract Targeting Cell Adhesion Proteins of Food-Borne Bacteria as a Potential Mechanism: Combined In Vitro-In Silico Approach. Plants.

[52] Kumar C.G., Mamidyala S.K. (2011). Extracellular synthesis of silver nanoparticles using culture supernatant of *Pseudomonas aeruginosa*. Colloids Surfaces. B Biointerfaces.

[53] Yamal G., Sharmila P., Rao K.S., Pardha-Saradhi P. (2013). Inbuilt Potential of YEM Medium and Its Constituents to Generate Ag/Ag_2_O Nanoparticles. PLoS ONE.

[54] Selvi B., Madhavan J., Santhanam A. (2016). Cytotoxic effect of silver nanoparticles synthesized from *Padina tetrastromatica* on breast cancer cell line. Adv. Nat. Sci. Nanosci. Nanotechnol..

[55] Manikandan R., Beulaja M., Thiagarajan R., Palanisamy S., Goutham G., Koodalingam A., Prabhu N.M., Kannapiran E., Basu M.J., Arulvasu C. (2017). Biosynthesis of silver nanoparticles using aqueous extract of *Phyllanthus acidus* L. fruits and characterization of its anti-inflammatory effect against H_2_O_2_ exposed rat peritoneal macrophages. Process Biochem..

[56] Elbeshehy E.K., Elazzazy A.M., Aggelis G. (2015). Silver nanoparticles synthesis mediated by new isolates of *Bacillus* spp., nanoparticle characterization and their activity against Bean Yellow Mosaic Virus and human pathogens. Front. Microbiol..

[57] Mahmoud W.M., Abdelmoneim T.S., Elazzazy A.M. (2016). The Impact of Silver Nanoparticles Produced by *Bacillus pumilus* as Antimicrobial and Nematicide. Front. Microbiol..

[58] Elkhalifa A.E.O., Al-Shammari E., Alam M.J., Alcantara J.C., Khan M.A., Eltoum N.E., Ashraf S.A. (2021). Okra-Derived Dietary Carotenoid Lutein against Breast Cancer, with an Approach towards Developing a Nutraceutical Product: A Meta-Analysis Study. JPRI.

[59] Coghlin C., Murray G.I. (2010). Current and emerging concepts in tumour metastasis. J. Pathol..

[60] Lee Y.C., Lin H.H., Hsu C.H., Wang C.J., Chiang T.A., Chen J.H. (2010). Inhibitory effects of andrographolide on migration and invasion in human non-small cell lung cancer A549 cells via down-regulation of PI3K/Akt signaling pathway. Eur. J. Pharmacol..

[61] Liotta L.A. (2001). An attractive force in metastasis. Nature.

[62] Jamal M., Ahmad W., Andleeb S., Jalil F., Imran M., Nawaz M.A., Hussain T., Ali M., Rafiq M., Kamil M.A. (2018). Bacterial biofilm and associated infections. J. Chin. Med. Assoc. JCMA.

[63] Adnan M., Patel M., Deshpande S., Alreshidi M., Siddiqui A.J., Reddy M.N., Emira N., De Feo V. (2020). Effect of Adiantum philippense Extract on Biofilm Formation, Adhesion with Its Antibacterial Activities against Foodborne Pathogens, and Characterization of Bioactive Metabolites: An in vitro-in silico Approach. Front. Microbiol..

[64] Barzegari A., Kheyrolahzadeh K., Hosseiniyan Khatibi S.M., Sharifi S., Memar M.Y., Zununi Vahed S. (2020). The Battle of Probiotics and Their Derivatives against Biofilms. Infect. Drug Resist..

[65] Upadhyay A., Upadhyaya I., Kollanoor-Johny A., Venkitanarayanan K. (2013). Antibiofilm effect of plant derived antimicrobials on Listeria monocytogenes. Food Microbiol..

[66] Onbas T., Osmanagaoglu O., Kiran F. (2019). Potential Properties of *Lactobacillus plantarum* F-10 as a Bio-control Strategy for Wound Infections. Probiotics Antimicrob. Proteins.

[67] García-Contreras R., Nuñez-López L., Jasso-Chávez R., Kwan B.W., Belmont J.A., Rangel-Vega A., Maeda T., Wood T.K. (2015). Quorum sensing enhancement of the stress response promotes resistance to quorum quenching and prevents social cheating. ISME J..

[68] Sybiya Vasantha Packiavathy I.A., Agilandeswari P., Musthafa K.S., Karutha Pandian S., Veera Ravi A. (2012). Antibiofilm and quorum sensing inhibitory potential of Cuminum cyminum and its secondary metabolite methyl eugenol against Gram negative bacterial pathogens. Food Res. Int..

[69] Husain F.M., Ahmad I. (2013). Doxycycline interferes with quorum sensing-mediated virulence factors and biofilm formation in gram-negative bacteria. World J. Microbiol. Biotechnol..

[70] Adonizio A., Kong K.F., Mathee K. (2008). Inhibition of quorum sensing-controlled virulence factor production in *Pseudomonas aeruginosa* by South Florida plant extracts. Antimicrob. Agents Chemother..

[71] Kessler E., Safrin M., Olson J.C., Ohman D.E. (1993). Secreted LasA of *Pseudomonas aeruginosa* is a staphylolytic protease. J. Biol. Chem..

[72] Winstanley C., Fothergill J.L. (2009). The role of quorum sensing in chronic cystic fibrosis *Pseudomonas aeruginosa* infections. FEMS Microbiol. Lett..

[73] Vattem D.A., Mihalik K., Crixell S.H., McLean R.J. (2007). Dietary phytochemicals as quorum sensing inhibitors. Fitoterapia.

[74] Falagas M.E., Makris G.C. (2009). Probiotic bacteria and biosurfactants for nosocomial infection control: A hypothesis. J. Hosp. Infect..

[75] Sadeghi B., Rostami A., Momeni S.S. (2015). Facile green synthesis of silver nanoparticles using seed aqueous extract of *Pistacia atlantica* and its antibacterial activity. Spectrochim. Acta. Part A Mol. Biomol. Spectrosc..

[76] Krishnaraj C., Jagan E.G., Rajasekar S., Selvakumar P., Kalaichelvan P.T., Mohan N. (2010). Synthesis of silver nanoparticles using *Acalypha indica* leaf extracts and its antibacterial activity against water borne pathogens. Colloids Surfaces. B Biointerfaces.

[77] Saxena A., Tripathi R.M., Zafar F., Singh P. (2012). Green synthesis of silver nanoparticles using aqueous solution of *Ficus benghalensis* leaf extract and characterization of their antibacterial activity. Mater. Lett..

[78] Awwad A., Salem N. (2012). Green Synthesis of Silver Nanoparticles by Mulberry Leaves Extract. J. Nanosci. Nanotechnol..

[79] Vidhu V.K., Aromal S.A., Philip D. (2011). Green synthesis of silver nanoparticles using *Macrotyloma uniflorum*. Spectrochim. Acta. Part A Mol. Biomol. Spectrosc..

[80] Bar H., Bhui D.K., Sahoo G.P., Sarkar P., Pyne S., Misra A. (2009). Green synthesis of silver nanoparticles using seed extract of *Jatropha curcas*. Colloids Surf. A Physicochem. Eng. Asp..

[81] Luna C., Chávez V.H., Barriga-Castro E.D., Núñez N.O., Mendoza-Reséndez R. (2015). Biosynthesis of silver fine particles and particles decorated with nanoparticles using the extract of *Illicium verum* (star anise) seeds. Spectrochim. Acta. Part A Mol. Biomol. Spectrosc..

[82] Da Cruz Paula A. (2016). Characterization of Different Breast Cancer Stem Cell Phenotypes in Proliferative, Pre-malignant and Neoplastic Lesions of the Breast: Associations with Breast Cancer Behavior and Progression. Ph.D. Thesis.

[83] El-Naggar N.E.-A., Hussein M.H., El-Sawah A.A. (2017). Bio-fabrication of silver nanoparticles by phycocyanin, characterization, in vitro anticancer activity against breast cancer cell line and in vivo cytotxicity. Sci. Rep..

[84] Reddy M.N., Adnan M., Alreshidi M.M., Saeed M., Patel M. (2020). Evaluation of Anticancer, Antibacterial and Antioxidant Properties of a Medicinally Treasured Fern *Tectaria coadunata* with its Phytoconstituents Analysis by HR-LCMS. Anti-Cancer Agents Med. Chem..

[85] Adnan M., Siddiqui A.J. (2021). Deciphering the Molecular Mechanism Responsible for Efficiently Inhibiting Metastasis of Human Non-Small Cell Lung and Colorectal Cancer Cells Targeting the Matrix Metalloproteinases by *Selaginella repanda*. Plants.

[86] Wu J.-G., Ma L., Lin S.-H., Wu Y.-B., Yi J., Yang B.-J., Wu J.-Z., Wong K.-H. (2017). Anticancer and anti-angiogenic activities of extract from *Actinidia eriantha* Benth root. J. Ethnopharmacol..

[87] Wiegand I., Hilpert K., Hancock R.E. (2008). Agar and broth dilution methods to determine the minimal inhibitory concentration (MIC) of antimicrobial substances. Nat. Protoc..

[88] Husain F.M., Ahmad I., Al-Thubiani A.S., Abulreesh H.H., AlHazza I.M., Aqil F. (2017). Leaf Extracts of *Mangifera indica* L. Inhibit Quorum Sensing—Regulated Production of Virulence Factors and Biofilm in Test Bacteria. Front. Microbiol..

[89] Blosser R.S., Gray K.M. (2000). Extraction of violacein from *Chromobacterium violaceum* provides a new quantitative bioassay for N-acyl homoserine lactone autoinducers. J. Microbiol. Methods.

[90] Taghadosi R., Shakibaie M.R., Masoumi S. (2015). Biochemical detection of N-Acyl homoserine lactone from biofilm-forming uropathogenic *Escherichia coli* isolated from urinary tract infection samples. Rep. Biochem. Mol. Biol..

[91] Essar D.W., Eberly L., Hadero A., Crawford I.P. (1990). Identification and characterization of genes for a second anthranilate synthase in *Pseudomonas aeruginosa*: Interchangeability of the two anthranilate synthases and evolutionary implications. J. Bacteriol..

[92] Huston A.L., Methe B., Deming J.W. (2004). Purification, characterization, and sequencing of an extracellular cold-active aminopeptidase produced by marine psychrophile *Colwellia psychrerythraea* strain 34H. Appl. Environ. Microbiol..

[93] Venkatramanan M., Sankar Ganesh P., Senthil R., Akshay J., Veera Ravi A., Langeswaran K., Vadivelu J., Nagarajan S., Rajendran K., Shankar E.M. (2020). Inhibition of Quorum Sensing and Biofilm Formation in *Chromobacterium violaceum* by Fruit Extracts of *Passiflora edulis*. ACS Omega.

[94] Musthafa K.S., Ravi A.V., Annapoorani A., Packiavathy I.S., Pandian S.K. (2010). Evaluation of anti-quorum-sensing activity of edible plants and fruits through inhibition of the N-acyl-homoserine lactone system in *Chromobacterium violaceum* and *Pseudomonas aeruginosa*. Chemotherapy.

[95] Ruch R.J., Cheng S.J., Klaunig J.E. (1989). Prevention of cytotoxicity and inhibition of intercellular communication by antioxidant catechins isolated from Chinese green tea. Carcinogenesis.

[96] Ashraf S.A., Al-Shammari E., Hussain T., Tajuddin S., Panda B.P. (2017). In-vitro antimicrobial activity and identification of bioactive components using GC-MS of commercially available essential oils in Saudi Arabia. J. Food Sci. Technol..

